# Managing energy transition alongside environmental protection by making use of AI-led butanol powered SI engine optimization in compliance with SDGs

**DOI:** 10.1016/j.heliyon.2024.e29698

**Published:** 2024-04-18

**Authors:** Muhammad Ali Ijaz Malik, Muhammad Usman, Muhammad Waqas Rafique, Sohaib Raza, Muhammad Wajid Saleem, Naseem Abbas, Uzair Sajjad, Khalid Hamid, Mohammad Rezaul Karim, Md Abul Kalam

**Affiliations:** a School of Civil and Environmental Engineering, FEIT, University of Technology Sydney, NSW, 2007, Australia; b Mechanical Engineering Department, Main Campus, University of Engineering and Technology Lahore, Pakistan; c Department of Mechanical and Energy Engineering, De Montfort University Dubai, United Arab Emirates; d Department of Mechanical Engineering, Sejong University, Gwangjin-gu, Seoul, 05006, South Korea; e Department of Energy and Refrigerating Air-Conditioning Engineering, National Taipei University of Technology, Taipei, 10608, Taiwan; f Department of Energy and Process Engineering, Norwegian University of Science and Technology, 7491, Trondheim, Norway; g Department of Mechanical Engineering, College of Engineering, King Saud University, Riyadh, 11421, Saudi Arabia

**Keywords:** Butanol-gasoline blends, Artificial intelligence, Statistical approach, Engine performance, Optimization

## Abstract

Enormous consumption of fossil fuel resources has risked energy accessibility in the upcoming years. The price fluctuation and depletion rate of fossil fuels instigate the urgent need for searching their reliable substitute. The current study tries to address these issues by presenting butanol as a replacement for gasoline in SI engines at various speeds and loading conditions. The emission and performance parameters were ascertained for eight distinct butanol-gasoline fuel blends. The oxygenated butanol substantially increases engine efficiency and boosts power with lower fuel consumption. The carbon emissions were also observed to be lower in comparison with gasoline. Furthermore, the Artificial Intelligence (AI) approach was used in predicting engine performance running on the butanol blends. The correlation coefficients for the data training, validation, and testing were found to be 0.99986, 0.99942, and 0.99872, respectively. It was confirmed that the ANN predicted results were in accordance with the established statistical criteria. ANN was paired with Response Surface Methodology (RSM) technique to comprehend the influence of the sole design parameters along with their statistical interactions controlling the responses. Similarly, the R^2^ value of responses in case of RSM were close to unity and mean relative errors (MRE) were confined under specified range. A comparative study between ANN and RSM models unveiled that the ANN model should be preferred. Therefore, a joint utilization of the RSM and ANN can be more effective for reliable statistical interactions and predictions.

## Highlights

• Artificial Intelligence driven engine optimization for butanol-gasoline blends.
• Improvement in engine performance including lower BSFC and higher BTE.
• Significant emissions reduction in CO and HC contents.
• ANN outperforms RSM in accurate predictions with MRE less than 2.96 %.
• Compliance with Sustainable Development Goals under United Nations Vision 2030.

## Graphical abstract

**Figure:**
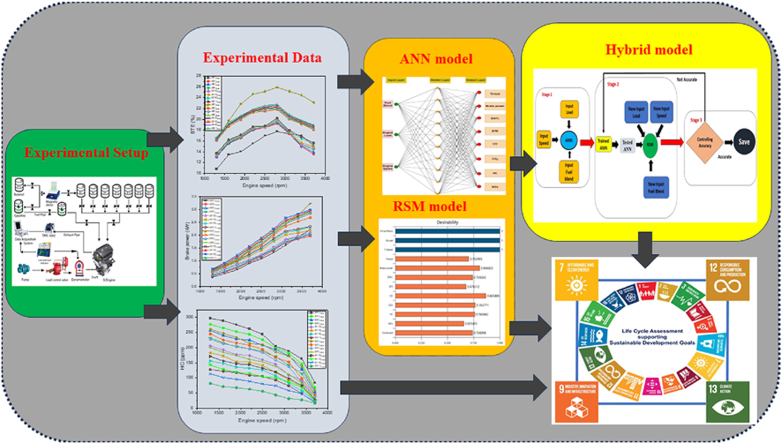

**Table:** Nomenclature:

AI	Artificial Intelligence	HC	Hydrocarbon
ANN	Artificial Neural Network	HRR	Heat release rate
ASTM	American Society for Testing Materials	ICP	In cylinder pressure
(A/F)_stoic_	Stoichiometric Air to Fuel ratio	LHV	Latent heat of vaporization
BP	Brake Power	MRE	Mean relative error
BSFC	Brake Specific Fuel Consumption	NOx	Oxides of nitrogen
BTE	Brake Thermal Efficiency	Ppm	Parts per million
CI	Compression Ignition	Psi	Pound per square inch
CR	Compression Ratio	RMSE	Root Mean Square Error
C/H	Carbon to Hydrogen ratio	RON	Research Octane Number
CO_2_	Carbon Dioxide	RSM	Response Surface Methodology
CO	Carbon Monoxide	SI	Spark Ignition
EGA	Exhaust Gas Analyzer	VE	Volumetric Efficiency
EGR	Exhaust Gas Recirculation	WOT	Wide Open throttle

## 1 Introduction

The advancements in human civilization and technology are happening at a tremendous pace. These advances come at the expense of significant energy consumption (14,243 Mtoe in 2020 to 17,487 Mtoe in 2040 an increase of 22.77 %) [1]. The human civilization is paying a steep price to fulfill energy requirements by using conventional fuels and power sources [2]. The projected timeframe for the depletion of fossil fuels is 50 years at the current rate of consumption [3]. The swift dwindling of conventional fuel reserves and alarming environmental threats emphasize serious efforts to search alternatives for gasoline. The pre-requisites in searching alternative fuels involve attention to such substitutes which are not only compatible with the engines but can also improve performance, reduce emissions, and are renewable in nature [4]. Biofuels are gaining significance owing to their renewable nature, biodegradable attributes, and ability to minimize air pollution [5]. As per estimates, 95 % of vehicles in the world meet their energy needs from fossil fuels and the transport sector contributes in 60–80 % of hazardous emissions [6]. The trend is rapidly shifting from conventional fuels to renewable fuels, as their share in the final energy consumption was 13 % in 2012, and projected to increase about 24 % in 2030 [7]. Biofuels mainly include alcohol, vegetable oils, and biodiesel. However, alcohols (oxygenated fuels) are widely used in IC engines owing to their ability to lower emissions (toxic and greenhouse), biodegradability, boost engine efficiency, and saving of fuel cost [8,9]. Alcoholic fuels are not only compatible with gasoline fuel but also used with diesel fuel as blended fuel. Seesy et al. [10] used methanol as blended fuel in diesel engine along with n-decanol as cosolvent in order to avoid fuel un-stability issues. Zhang et al. [11] employed n-butanol as co-solvent to improve mutual stability of ethanol and diesel fuel. Among other alcoholic fuels, butanol is less susceptible to moisture contamination (immiscible in water) and ensures phase stability. Butanol exhibits lower vapor pressure and latent heat vaporization (LHV) along with higher calorific value, as these characteristics responsible for its more viability in comparison with other alcoholic fuels [12]. Butanol has a longer carbon chain than both methanol and ethanol. This structural difference can lead to improved combustion characteristics and better compatibility with the existing engines, making it an attractive option for automotive applications [13]. Moreover, butanol is less toxic than methanol, which can be hazardous if ingested or inhaled. The lower toxicity enhances its usage and favor its choice in certain applications [14]. Many studies suggested that butanol produced the least CO_2_ and NOx emissions, higher brake thermal efficiency and lower fuel consumption in comparison to other alcoholic fuels [[14], [15], [16], [17], [18], [19]].

The higher flash point and boiling temperature of butanol make it secure for storage and transportation [20]. The butanol is projected to be a viable and sustainable alternative fuel after series of investigations over the past years [21]. Butanol blends create positive impact on HC and CO emission owing to higher oxygen to fuel ratio while lower C/H and (A/F)_s_ ratios and fast flame speed in case of butanol [22,23]. It can be deduced that operating conditions such as A/F ratio, engine speed, loading, ignition timing significantly affect carbon emissions. Mixed literature exists for NOx emissions when engine fueled with butanol blends. The increase in NOx contents for butanol blends credited to their higher anti-knock ability, oxygen content and quicker flame propagation. The factors like spark timing, EGR rate, temperature (cylinder and ambient), engine speed, load and A/F ratio impact NOx emission [23,24]. While in some cases, NOx emission decreases for butanol blends. This decline in the NOx emission can be reasoned with the higher lower heating value (LHV) and hydroxyl (OH) group that reduced the CH radicals [25]. The higher LHV of butanol is responsible for a higher volumetric efficiency (VE) and mean effective pressure, which as consequence increases the output torque and power [26,27]. Butanol possesses lower calorific value in comparison with the gasoline which mainly responsible for higher brake specific fuel consumption (BSFC) [23]. But in many cases, compression ratio, brake torque and spark timing are mainly responsible for the lower BSFC in case of butanol [20]. Elfasakhany [28] observed that at lower engine speed hydrocarbon (HC), carbon dioxide (CO_2_) and carbon monoxide (CO) emission for the butanol blends were 26 %, 43 % and 32 % decremented than of gasoline. While at higher engine speed CO_2_ for the blended fuel increased by 27 %, but the CO and HC emission declined by 6 and 11 %, respectively. Yusuf et, al [29]. recorded that BSFC gradually declined by 3.79 % for the butanol blended fuel, resultantly the combustion rate improved by 50 % and reduced ignition delay with rise in in-cylinder pressure (ICP) and heat release rate (HRR). Huynh [30] found that BSFC for B20 declined by 3.6 % and 8.4 % for 2250 rpm and 4250 rpm under 30 % wide open throttle (WOT) condition. At 70 % WOT condition, it declined by 1.5 and 4.3 % for B20 at 2250 and 4250 rpm respectively. Furthermore, the NOx emission for the B25 increased by 22 and 57 % for 2250 and 4250 rpm condition, respectively under 30 % WOT condition. While at 70 % WOT condition, the NOx increased by 33 and 52 % at 2250 and 4250 rpm respectively. Hussain et al. [31] conducted experiment to evaluate engine performance for the B6 and B12 fuel blends. They observed a 7.50 and 12.45 % increase in torque for the B6 and B12 fuel blends as compared to gasoline (B0). Also, the brake thermal efficiency (BTE) increased by 2.12 % and 3.25 % for B6 and B12 respectively in comparison with B0. While B12 demonstrated a 12.5 % rise in brake power (BP) in contrast with base fuel.

On the other hand, the utilization of artificial intelligence techniques to predict experimental results is growing very quickly due to cost and time associated with experimentation. Response surface methodology (RSM) and Artificial neural network (ANN) approaches are effectively replaced classical modeling approaches for solving engineering problems. The predictability of ANN model depends on the experimental data training followed by testing and validation. If predicted data is undesirable, then ANN may re-train data to improve performance. On this note, the ANN models have been extensively used in predicting the IC engines parameters. Ahmed et al. [32] compared experimental results with the ANN predicted model for methanol blended fuels. The mean relative error (MRE) was obtained in between 1.2 and 2.4 % and regression correlation coefficient (R) was obtained in between 0.9910 and 0.9983. Sayin et al. [33] attained R in range of 0.983–0.99 through ANN modeling of engine. Similarly, Kiani et al. [34] found R value in between 0.71 and 0.99 for the ethanol blended fuels. Kapusuz et al. [35] also predicted engine performance for the ethanol-methanol blends and deduced that best performance achieved for blend M11E1 (Methanol 11 %, Ethanol 1 %) and the R was in between 0.931 and 0.990. Likewise, RSM is a statistical regression technique to predict and optimize results. RSM was used by Ardebili [36] to examine the performance of SI engines running on different alcoholic fuel blends (0–100 % with a gap of 25 %) under various loading situations (20 %, 40 %, 60 %, 80 %, and 100 %) at a fixed speed of 2500 rpm. The optimization findings suggest a 47.21 % engine load and a 25 % fusel oil level. At these optimum operating circumstances, the torque (16.49 N-m), CO (0.88 %), HC (165.49 ppm), BSFC (326.02 g/kWh), and NOx (568.3 ppm) were measured. Along with significantly reduced in-cylinder temperatures, the BTE and torque decreased. For alcoholic concentration rose from 20 to 100 %, 41 % decline in NOx contents, and 22 % rise in CO, and 39 % rise in HC contents were achieved. Usman et al. [37] conducted experiment on diesel-HHO mixture by employing ANN coupled with RSM to predict BTE and BSFC. In the case of BTE, the RSM and ANN predicted MRE was 2.26 and 1.91 % respectively. However, the RSM and ANN predicted MRE in case of BSFC was 2.64 and 2.94 %. Likewise, root mean square error (RMSE) by RSM and ANN in case of BSFC were 0.088 and 0.012 kg/kWh respectively.

In the pursuit of improving engine performance and mitigating emissions, researchers have explored the intriguing domain of fuel blends incorporating butanol and gasoline. This innovative study aims to unravel the intricate dynamics between butanol and gasoline when utilized as a blended fuel in internal combustion engines. The investigation is made to shed light on the potential synergies that could redefine the landscape of sustainable fuel solutions. It can be observed from the literature that ANN assisted RSM model has not yet been developed for butanol-gasoline blends. The current study integrates cutting-edge techniques such as Artificial Neural Networks (ANN) and Response Surface Methodology (RSM) for the optimization of engine performance. ANN predicted results than optimized by RSM followed by the validation through experimentation. The ANN predicted results used as input data to RSM and then RSM provide optimized results which are then validated through experimentation. The absolute percentage error turned to be below 4 % which is an indicator of the authenticity of RSM. This groundbreaking approach harnesses the power of artificial intelligence and statistical modeling to unlock the full potential of butanol-gasoline blends while paving the way for the cleaner and more efficient combustion processes. As the automotive industry stands at the cusp of transformation, this study stands as a beacon, illuminating the path towards greener and more sustainable transportation solutions.

## 2 Methodology

In current work, the SI engine (HONDA GP160) operated on four strokes with air cooling mechanism was used to ascertain its performance and emissions when operated on different butanol-gasoline blends. Table 1 represents characteristics of engine used in the current study.

**Table 1:** SI engine attributes.

Parameters	Units	Characteristics
Bore	m	0.0068
Displacement	cm^3^	163
Max torque	N-m	10.3
Number of vales	–	2
Stroke	m	0.0045
Net power	kW	3.6
Fuel tank storage	L	3.1
Compression ratio	–	8.5/1
Engine operating hours prior experiment	h	170
Lubricant oil storage	L	1.6

### 2.1 Test fuels

A gasoline as base fuel (B0) was attained from Pakistan State Oil (PSO). While the Butanol (B100) was attained from the Merck chemicals. The gasoline and butanol were blended according to percent by volume (%v) like 3 % butanol blended in 97 % gasoline (B3), 6 % butanol blended in 94 % gasoline (B6), 9 % butanol blended in 91 % gasoline (B9), 12 % butanol blended in 88 % gasoline (B12), 15 % butanol blended in 85 % gasoline (B15), 18 % butanol blended in 82 % gasoline (B18) and 21 % butanol mixed in 79 % gasoline (B21), with the objective to determine the performance and exhaust emission parameters of the SI engine. Both gasoline and butanol were primarily blended in measuring chamber with reference to their %v, then the blended fuels were allowed to be mixed simultaneously with the help of magnetic stirrer for 45 min to ensure homogenous mixing of the test fuels. The titration method guarantees the perfect composition of blends in the magnetic stirrer. After magnetic stirring, the fuel blends were filled in a container for two days to allow homogenous mixing of fuel blends before being employed into the engine. Furthermore, the fuel storage containers are kept in the moisture free zone to avoid any phase separation owing to moisture. The eight test fuels (B0, B3, B6, B9, B12, B15, B18 and B21) were fueled into engine intake manifold through 500 mL transparent measuring cylinder. The measuring cylinder has the least count of 1 mL for fuel flow rate calculation. Eight samples of test fuels, with distinct ratios of butanol in gasoline as blend with attributes as stated in Table 2 were employed.

**Table 2:** Physicochemical attributes of eight test fuels.

Fuel characteristics	Density	Calorific value	Oxygen content	(A/F) _stoic_	LHV	Octane rating	Kinematic viscosity at 20 °C
Units	kg/m³	MJ/kg	% v/v	kg/kg	kJ/kg	–	mm^2^/s
Standard	ASTMD1298	ASTM D240	ASTM D5622	SAE J1829-201503	ASTM D323	ASTM D2699	ASTM D445
B0	731	44.04	0	14.7	300	92	0.76
B3	734	43.59	0.64	14.5	308.4	92.15	0.82
B6	737	43.14	1.29	14.4	316.9	92.28	0.87
B9	740	42.70	1.94	14.38	325.3	92.39	0.93
B12	743	42.25	2.59	14.2	333. 8	92.48	0.98
B15	746	41.81	3.24	14.1	342.3	92.63	1.04
B18	749	41.36	3.88	14.0	350.7	92.75	1.16
B21	752	40. 92	4.53	13.9	359.2	92.87	1.23
B100	831	29.2	21.6	11.2	582	96	2.51

### 2.2 Experimental setup

**Fig. 1:**
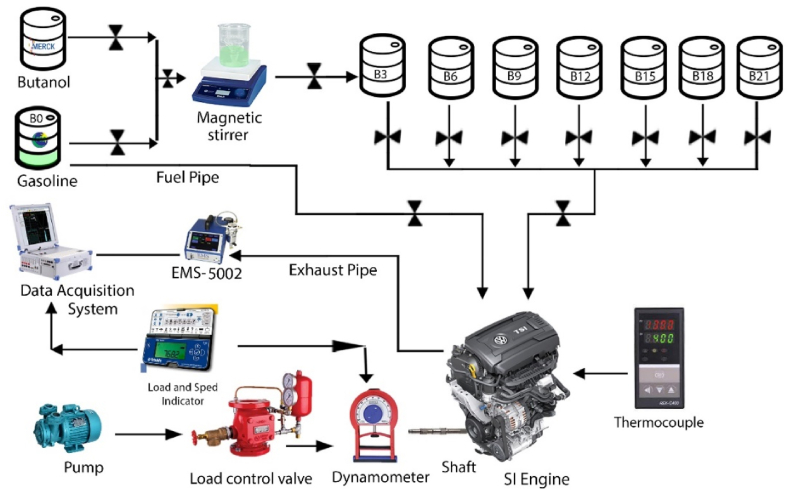
Experimental setup.

Fig. 1 explains schematic arrangement of all the components used in experimentation. The engine is connected with dynamometer, emission analyzer and fuel intake gauge as depicted in Fig. 1. An experiment was accomplished on a 4 stroke, carburetor type SI engine having single cylinder and 163 cm^3^ displacement. The fuel from fuel intake gauge and air from intake manifold mixed in carburetor and then the mixture delivered into engine cylinder for burning. All the eight test fuels were filled into the engine via transparent measuring gauge of 500 mL capacity with least count of 1 mL for the measurement of fuel consumption. The BSFC was measured through the ratio between the mass of fuel supplied and the brake power obtained through the burning of a particular fuel blend. The mass of fuel supplied is determined by calculating the time for the 300 mL consumption of fuel and then multiplied with the specific gravity of the particular fuel blend. On the other hand, the brake torque was evaluated by water brake dynamometer of Dynomite company by varying speed according to SAE J1349 standard. The laptop installed with DYNO-MAX 2010 software was turned to be data acquisition system. The exhaust gases (HC, CO, NOx, and CO_2_) were recorded through an exhaust gas analyzer modeled as EMS-5002. The 1 horsepower (hp) pump was linked with dynamometer, used to vary water pressure which ultimately varies load on engine. The dynamometer receives water from pump through load control valve, the water lashes around tiny toroidal pockets inside the casing. The shear forces in pressurized water directed tangential to housing radius in opposite direction of engine shaft motion, ultimately act as load.

### 2.3 Test scheme

The test scheme designed for current investigation is stated in Table 3. Before experimentation, the engine was allowed to warm up for 15 min and engine seals were examined to prevent any leakage, while fresh air filters were employed for proper oil and air supply. Tests were initiated at 1300 rpm and terminated at 3700 rpm with an equal gap of 300 rpm at each stage. The emission parameters along with engine performance were evaluated at distinct nine engine speeds, two different loads and eight different fuel blends. Accounting each feasible combination of constantly fluctuating factors, 144 distinct experimental readings were noticed. The fuel consumption was ascertained by gauging fuel decline with reference to time elapsed at each speed and loading condition. EMS-5002 probe was introduced in exhaust pipe and allowed to be there for 1 min at each loading and speed condition. Finally, the engine performance was modeled as predicted through ANN. The engine performance than optimized through RSM technique along with desirability aspect of each response parameter.

**Table 3:** Comprehensive experimental scheme.

Testing parameters	Details
Speed range	1300:300:3700 rpm
Fuel	B0: B3: B21
Loading conditions	15 and 30 psi
Performance parameters	Torque, BTE, BP, and BSFC
Emission parameters	CO_2_, NOx, CO, HC,
Atmospheric temperature	22 °C
Ambient pressure	101325 Pa

### 2.4 Uncertainty analysis

The Uncertainty analysis allows for determining the accuracy of measured parameters and provides information about the magnitude of error associated with each measurement in the experimental setup. Table 4 entails the accuracy, range and uncertainty linked with the measured parameters. The overall uncertainty of experimental setup (U_exp_) is determined with the following equation [32]. The overall uncertainty turned out to be 2.34 %, so the engine performance is believed to be accurate within ±3 %.

$$U_{\exp\;}={\left[\;{\left(U_{\text{HC}}\right)}^{2\;}+{\left(U_{\text{Speed}}\right)}^{2\;}+{\left(U_{\text{NOx}}\right)}^{2\;}+{\left(U_{\text{Power}}\right)}^{2\;}+{\left(U_{\text{CO}}\right)}^{2\;}+{\left(U_{\text{CO}2}\right)}^{2\;}+{\left(U_{\text{FC}}\right)}^{2\;}\right]}^{1/2}$$

$$U_{\exp\;}={\left[\;{\left(1\right)}^{2\;}+{\left(0.5\right)}^{2\;}+{\left(1\right)}^{2\;}+{\left(1\right)}^{2\;}+{\left(1\right)}^{2\;}+{\left(1\right)}^{2\;}+{\left(0.5\right)}^{2}\right]}^{1/2}$$

$$U_{\exp\;}=2.34\%$$

**Table 4:** Accuracy and range of instruments for measured parameters.

Measured parameters	Accuracy	Range	Uncertainty (%)
NO_x_	±1 ppm	0–5000 ppm	±1
Speed	±5 rpm	0–8000 rpm	±0.5
CO	±0.01 %	0–18 %	±1
Fuel consumption	±0.1 mL	0–500 mL	±0.5
HC	±1 ppm	0–5000 ppm	±1
Power	±0.05 kW	0–50 kW	±1
CO_2_	±0.1 %	0–18 %	±1
Torque	±0.1 N-m	0-45 N-m	±1

### 2.5 Artificial neural network

**Fig. 2:**
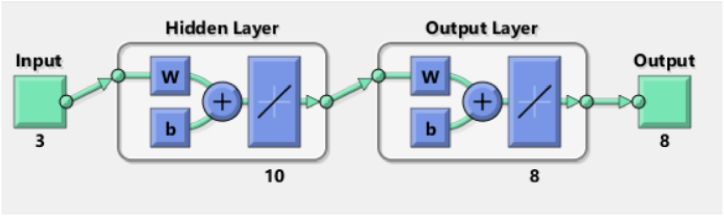
Feedforward back propagation ANN.

The artificial neural network (ANN) comprises an intersecting collection of artificial neurons, employing the connectionist method to process information [38]. Through this approach, the ANN establishes an analytical model to address projection and decision making challenges [39]. Each neuron receives input values to produce a conforming output behavior, which is stored in networks [33]. This behavior of neuron is then conveyed via interconnections to other neurons as input signals. The arrangement of the neural network, along with interpretation, regularization of the data, and output responses; significantly influences the effectiveness and functioning of the training of neural network [40]. In the conventional sense, ANN model consists of few mandatory layers: input, hidden, and the output layers. The role of first layer is to apply the target data, while the subsequent output data is obtained by output layer [39]. Neural operator learning occurs through a relationship among a specific set of input and output data in the hidden layer. This study utilized a feed-forward backpropagation (FFBP) network, where neurons are organized in layers, sending signals forward, but errors are propagated backward [38]. Neural networks acquire target data from the input layer, along with the output obtained from the output layer [41]. The FFBP algorithm is employed for controlling learning rate and to estimate errors [42], representing the change amid experimental (Ev) and predicted (Pv) values. The general ANN-FFBP network model in the present investigation is shown Fig. 2. Evaluation of the neural network involves minimizing the error, and training and testing are essential steps in this investigation. MATLAB/Simulink R2021a was used for the neural network training, specifically employing the 'nntool' module to predict combustion engine process parameters fueled with gasoline and butanol blended fuels. Input parameters include fuel blend, engine load, and speed, while torque, BP, BSFC, BTE, NOx, CO_2_, HC, and CO serve as output responses for training the neural networks. The weights between input and output layers, as well as between hidden and output layers, are randomly generated based on the designated topology of the neural network [38].

### 2.6 Response surface methodology

In the current work, the design of expert 11 is employed to construct a regression model. Within this framework, Response Surface Methodology (RSM) is utilized to finely optimize cutting factors [40]. The RSM model's validity is substantiated by a probability value (p-value) < 0.0001. A three-dimensional surface plot based on the effects of input control factors (fuel blend, engine load, and speed) and subsequent output factors (Torque, BP, BSFC, BTE, NOx, CO_2_, HC, and CO) is generated. In this investigation, the RSM is applied to optimize engine load and speed, aiming to achieve favorable engine characteristics for the gasoline engine fueled with the recommended butanol-blended fuels. The resulting model undergoes Analysis of Variance (ANOVA), and a regression analysis is conducted to derive coefficients and mathematical relationships that can predict output responses from experiments. The significance of a parameter is determined by evaluating its 'P' value, with a value below 0.05 indicating a 95 % confidence level [42]. Exploring the interactive impacts of engine operating factors and output responses involves employing statistical Design of Experiments (DOE) techniques [41]. Ultimately, input variables are optimized using the Desirability consideration within RSM, whereas solution combination having highest desirability is chosen as the optimal combination.

## 3 Results and discussion

The experimental data is first displayed as line graphs, then these experimental results are exported to the MATLAB/Simulink platform for the Artificial Neural Network prediction. These ANN predicted results compared with experimental results. Further, ANN predicted results exported to Design Expert software for the implementation of RSM. The predicted results (RSM) are then validated by experimentation.

### 3.1 Experimental results

**Fig. 3:**
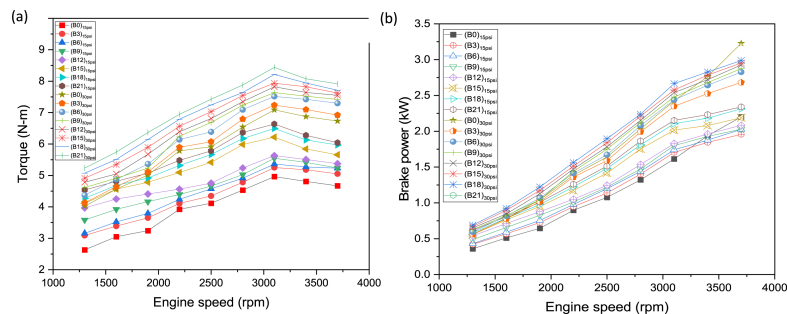
(a) Torque, and (b) BP variation wrt engine speed and load for butanol-gasoline fuel blends.

Torque is the keen factor that defines the operation ability of an engine under higher loading conditions. The change in torque for the distinct gasoline-butanol blends can be observed from Fig. 3(a). It is apparent that torque is significantly increased for the butanol blended fuels as compared to gasoline. This increased torque can be reasoned out with additional oxygen content and quicker propagation of laminar ﬂame in case of alcoholic oils [43]. It is clear from Fig. 3(a) that generally torque first rises when reached at maximum level then decline after that maximum point. This behavior can be accredited with the reason that when the speed is less then explosions due to burning of fuel are also less, which create less impact on piston to push it down and less force applied on the connecting rod, which might be responsible for less turning effect around the crankshaft. With the progression in engine speed, the optimum range was then reached with the peak efficiency transforming more energy from the fuel to generate higher torque. Further progression in speed resulted in weaker explosions due to engine breathing issues at higher speeds. The volumetric efficiency drop for all test fuels as speed increases, due to choking in flow and frictional losses at intake [44]. This drop in volumetric efficiency is mainly responsible for the decline in torque after certain rpm (3100). At 15psi loading condition B3 to B21 produced 8.11 %, 11.48 %, 16.72 %, 21.58 %, 32.64 %, 38.09 %, and 41.93 % respectively. At 30 psi loading condition B3, B6, B9, B12, B15, B18 and B21 produced 2.62 %, 7.49 %, 9.49 %, 12.45 %, 15.51 %, 18.54 %, 35.60 %. The blends B0:B3:B21 at higher loading condition produced 46.16 %, 38.73 %, 40.95 %, 37.09 %, 35.18 %, 27.27 %, 25.46 %, and 25.51 % more torque than B0:B3:B21 at lower loading condition. The augmented latent heat of vaporization for butanol blended fuel is responsible for the temperature drop of incoming charge and also in combustion chamber owing to evaporation. As a consequence, higher charge density engendered higher engine torque [45]. It is apparent from Fig. 3(b) that butanol blended fuels generated more brake power as compared to gasoline. Both fuels showed a similar growing trend in brake power as speed increases. The increased latent heat of vaporization when fusel oil is used in an engine ensured the incoming air into the engine cylinder cools down which resulted into higher charge densities, volumetric efficiencies, and power output [46,47]. The higher brake power for butanol blended fuels can be attributed to more laminar flame and octane rating of butanol [48]. At 15psi loading condition, B3 to B21 produced 1.40 %, 4.47 %, 8.30 %, 12.04 %, 22.16 %, 27.44 %, and 30.62 % more brake power than B0 respectively. At 30 psi loading condition B12, B15, B18 and B21 produced 2.24 %, 4.73 %, 7.27 % and 10.15 % more brake power than B0 respectively. But B3, B6, B9 produced 6.67 %, 1.96 %, 0.18 % less brake power than B0 respectively. The reasons behind the lower brake power in the case of B3, B6, and B9 compared to B0 at higher loading conditions may be due to the lower calorific value of butanol, higher octane rating of butanol which sometimes slower combustion rates, and lower flame speed. The blends B0:B3:B21 at higher loading condition produced 50.22 %, 38.72 %, 40.99 %, 37.09 %, 38.46 %, 37.08 %, 28.79 %, and 26.44 %, more brake power than B0:B3:B21 at lower loading condition. Butanol comprises of 21.6 % oxygen by mass and this attribute mainly responsible for lean mixture which in consequence improves combustion process and ultimately higher power obtained [49].

**Fig. 4:**
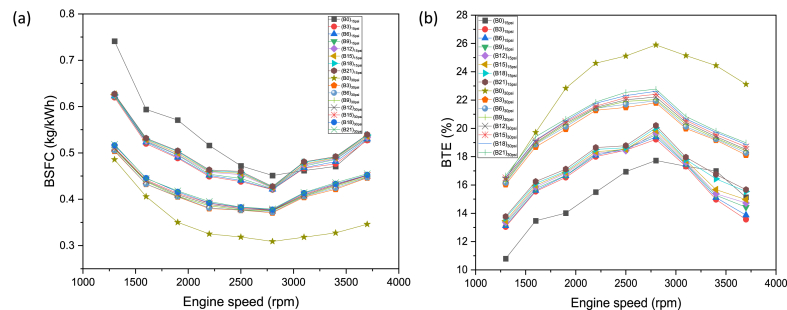
(a) BSFC, and (b) BTE trends wrt engine speed and load for butanol-gasoline fuel blends.

It can be observed from Fig. 4(a) that the general trend of BSFC remains the same for all test fuels. The BSFC remains higher at the start but gradually declines. At a certain point (2800 rpm), the BSFC exhibits minimum value and then starts increasing as the experiment proceeds further. Precisely, the more fuel is utilized by engines at the beginning to overcome inertial effects and to attain running state. The heat loss was more at lower engine rpm which mainly responsible for higher fuel consumption to recompense these losses [50,51]. The lowest BSFC at certain engine speed depicts stoichiometric combustion [50]. The BSFC starts rising as engine speed increases to compensate for higher power needs. The butanol-gasoline blends depict lower BSFC in comparison with gasoline due to higher fuel density and BMEP and ultimately resulting an increase in BTE [52,53]. At 15psi loading condition, B3, B6, B9, B12, B15, B18 and B21 produced 8.31 %, 7.84 %, 7.32 %, 6.83 %, 6.43 %, 6.11 %, and 5.82 % less BSFC than B0 respectively. At 30 psi loading condition B3, B6, B9, B12, B15, B18 and B21 produced 17.43 %, 17.80 %, 18.47 %, 19.07 %, 19.55 %, 20.18 % and 20.64 % more BSFC than B0 respectively. The blends B0:B3:B21 at higher loading condition exhibit 33.72 %, 15.11 %, 15.28 %, 15.26 %, 15.29 %, 15.31 %, 15.15 %, and 15.09 % lower BSFC than B0:B3:B21 at lower loading condition. The more BSFC at higher loading conditions owing to more fuel consumption to meet higher power requirement. BTE represents fraction of BP produced by engine with respect to input fuel energy. An overall BTE trend for all the eight test fuels is demonstrated in Fig. 4(b), which discloses that the BTE initially rises to max level, then the BTE eventually fell down. The BTE first rises as engine speed approaches towards 2800 rpm owing to lean mixture formation. At higher engine rpm, BTE declines swiftly due to abrupt combustion [54]. The thermodynamic second law postulates that the engine efficiency will increase if heat losses from engine decrease. Furthermore, the BTE possess inverse relation with calorific value and BSFC, therefore, any decline in heat loss and lower calorific value can boost the engine efficiency [55]. The increase in power along with effective fuel burning were among the significant aspects in augmenting the BTE in case of butanol blended fuels. At the 15psi loading condition, B3, B6, B9, B12, B15, B18 and B21 produced 0.97 %, 1.11 %, 1.26 %, 1.36 %, 1.51 %, 1.76 %, and 1.91 % more BTE than B0 respectively. At 30 psi loading condition B3, B6, B9, B12, B15, B18 and B21 produced 3.42 %, 3.27 %, 3.17 %, 3.06 %, 2.92 %, 2.79 % and 2.64 % less BTE than B0 respectively. The blends B0:B3:B21 at higher loading condition exhibit 7.71 %, 3.32 %, 3.33 %, 3.29 %, 3.30 %, 3.29 %, 3.17 %, and 3.16 % more BTE than B0:B3:B21 at lower loading condition. Higher latent heat of evaporation of blended fuel aids in vaporizing the fuel in compression stroke. In consequence, fuel captivates heat from cylinder walls and evaporates along with compression, thus boosting BTE [56]. Moreover, oxygen content in butanol aids in improving combustion and thermal efficiency [26].

**Fig. 5:**
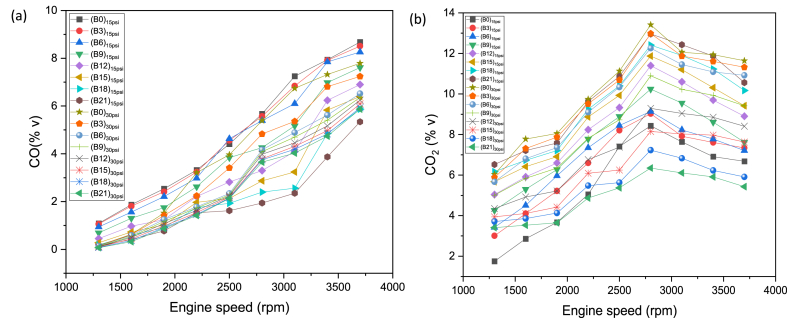
(a) CO, and (b) CO_2_ variation wrt engine speed and load for butanol-gasoline fuel blends.

CO is noxious gas which emanates as exhaust from engine owing to inappropriate proportion of air in air-fuel mixture [57]. Fig. 5(a) indicates variation in CO emissions for eight test fuels at nine speeds and two loading condition. It was noticed that mixing butanol in primary fuel results in a reduction in CO emissions. As, butanol is oxygenated fuel which mainly responsible for the leaning effect during fuel combustion [58]. At the 15psi loading condition, B3, B6, B9, B12, B15, B18 and B21 produced 2.01 %, 6.66 %, 19.99 %, 33.99 %, 42.78 %, 50.78 %, and 57.98 % less CO emission than B0 respectively. At 30 psi loading condition B3, B6, B9, B12, B15, B18 and B21 produced 12.66 %, 25.65 %, 28.10 %, 30.77 %, 33.52 %, 35.54 % and 37.09 % less CO emission than B0 respectively. The blends B0, B3, B6, B9, B12 and B15 at higher loading condition produced 14.15 %, 23.45 %, 31.61 %, 22.85 %, 9.95 % and 0.25 % less CO emission than B0, B3, B6, B9, B12 and B15 at lower loading condition. While B18 and B21 at higher loading condition produced 12.45 % and 28.55 % higher CO emission under lower loading condition. The lower CO emissions for the butanol-gasoline blends results coincides with the previous studies [24,59,60]. The combustion products under stoichiometric condition only comprised of CO_2_ and H_2_O. Owing to insufficient combustion and undesired substances fuel and air during their induction, there are other emissions as well. CO_2_ emission is directly associated with thermal efficiency, fuel consumption and torque. CO_2_ emission for eight test fuels under two distinct loading condition and various speeds is exhibited in Fig. 5(b). As observed from Fig. 5(b), the butanol blended fuels produced more CO_2_ emission than gasoline. The higher CO_2_ production can be reasoned out with more fuel consumption in case of blends, the presence of more carbon and oxygen produced more CO_2_ [61]. At 15psi loading condition, B3, B6, B9, B12, B15, B18 and B21 produced 17.14 %, 23.13 %, 35.95 %, 50.23 %, 59.92 %, 69.58 %, and 77.84 % higher CO_2_ emission than B0 respectively. At 30 psi loading condition B3, B6, B9, B12, B15, B18 and B21 produced 2.99 %, 7.40 %, 19.12 %, 30.04 %, 38.35 %, 46.61 % and 51.14 % less CO_2_ emission than B0 respectively. The blends B0, B3, B6 and B9 at higher loading condition produced 82.12 %, 50.81 %, 36.96 %, and 8.35 % higher CO_2_ emission than B0, B3, B6, and B9 at lower loading condition. While B12, B15, B18 and B21 at higher loading condition produced 15.19 %, 29.79 %, 42.66 % and 50.21 % lower CO_2_ emission than B12, B15, B18 and B21 at lower loading condition.

**Fig. 6:**
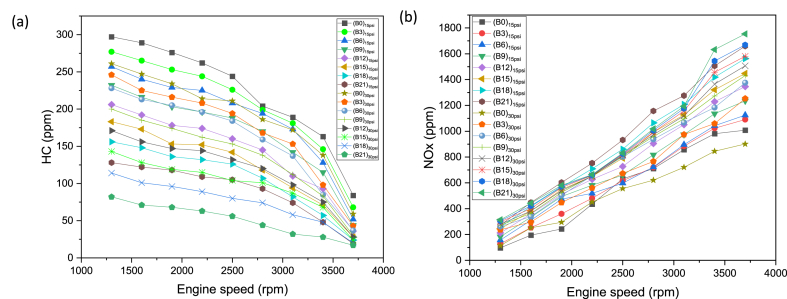
(a) HC, and (b) NOx variation wrt engine speed and load for butanol-gasoline fuel blends.

HC emission for all test fuels under distinct loading condition and speed range from 1300 to 3700 rpm can be observed from Fig. 6(a). HC emission generally declines with the rise in speed as depicted in Fig. 6(a). The general declining trend might be due to higher combustion rate [62]. The significant factors that impact the HC emission comprised of the exhaust valve leakage, engine misfire, unburned fuel accretion in crevices along with fuel state in the course of engine warmup [63,64]. The overall declining behavior of the HC emission obtained for blended fuels. It is credited to quicker flame propagation along with higher oxygen proportion in blended fuels [65]. The physicochemical properties of butanol blends aid in appropriate combustion and result decline in HC emissions [66]. Moreover, the accumulation of butanol decreases hydrocarbon proportion in fuel and eventually decrements HC emission [24]. At 15psi loading condition, B3, B6, B9, B12, B15, B18 and B21 produced 7.42 %, 15.04 %, 25 %, 35.56 %, 44.52 %, 51.79 % and 59.36 % lower HC emission than B0 respectively. At 30psi loading condition, B3, B6, B9, B12, B15, B18 and B21 produced 9.93 %, 16.14 %, 28.17 %, 37.81 %, 48.38 %, 60.51 % and 73.23 % lower HC emission than B0 respectively. The blends B0:B3:B21 at higher loading condition produced 14.24 %, 16.57 %, 15.36 %, 17.86 %, 17.23 %, 25 %, 29.75 %, 43.50 % lower HC emission than B0:B3:B21 at lower loading condition. The major proportion of air includes nitrogen and oxygen, therefore the oxides of nitrogen become significant during burning of air fuel mixture. Fig. 6(b) shows the NOx emission for all the eight test fuels at designated speeds and loading condition. Fig. 6(b) depicts that butanol blends produced more NOx emission than gasoline. This behavior can be reasoned with higher temperature, higher oxygen contents, and more air settling time in cylinder [67]. NOx emission usually rise with progression in load and speed due to higher fuel combustion and higher flame speed in order to meet higher power requirement [53,62]. At 15psi loading condition B3, B6, B9, B12, B15, B18 and B21 produced 7.75 %, 13.32 %, 24.39 %, 35.15 %, 57.81 %, 56.44 % and 66.85 % higher NOx emission than B0 respectively. At 30psi loading condition, B3, B6, B9, B12, B15, B18 and B21 produced 9.93 %, 16.14 %, 28.17 %, 37.81 %, 48.38 %, 60.51 % and 73.23 % lower HC emission than B0 respectively. The blends B3, B6, B9, B12, B15 and B18 at higher loading condition produced 12.77 %, 21.95 %, 15.11 %, 9.48 %, 5.59 % and 1.42 % higher NOx emission than B3, B6, B9, B12, B15 and B18 at lower loading condition. While B0 and B21 at higher loading condition produced 7.77 % and 2.02 % lower NOx emission than B0 and B21 at lower loading condition. The oxygenated fuel (butanol blends), makes fuel mixture lean and results to higher NOx emissions [68].

### 3.2 ANN model

**Fig. 7:**
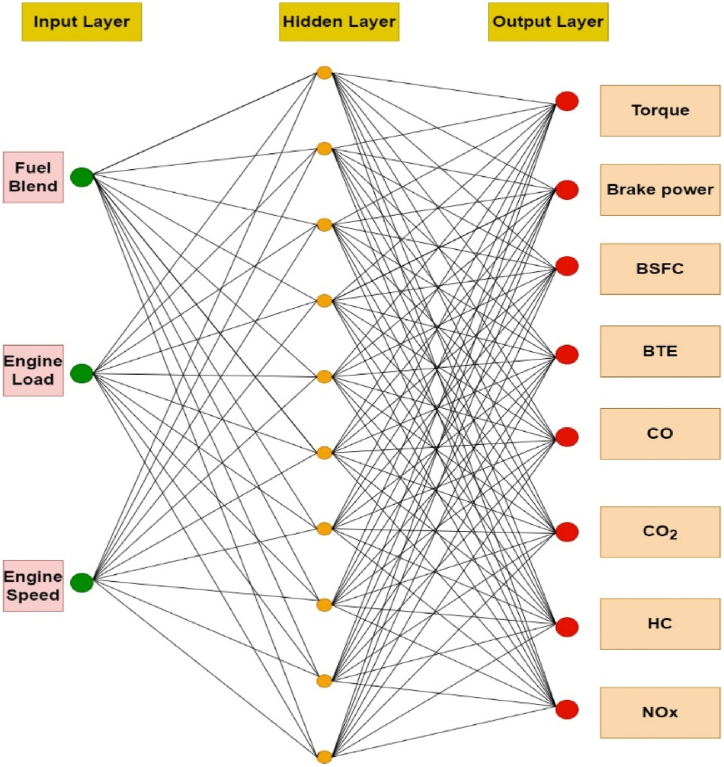
ANN model.

ANN model is mainly a statistical tool having a strong relevance with human brain processing system [37]. ANN is potent tool for analyzing, optimizing, and forecasting of non-linear data results. The ANN model has extensive applications in the automotive sector with most accurately estimated performance characteristics. Generally the ANN exhibits three distinct layers (input, hidden and output layer) comprised of processing neurons, but the number of layers can be increased depending upon intricacy of data [69,70]. Neurons carry information from one layer to other through connection weights and interlinked structure of weighted biases [71]. ANN model depends on experimental data which then trained, tested, and validated for output prediction under distinct situations. An activation function served as a function amid layers to predict response through trained data set. The conception of learning process comprised of the RMSE and MRE which is employed to enhance accuracy. The input layer comprised of the user-defined entities (experimental data). The ANN predicted variables generated after neuron processing through certain activation function. The data training involves iterations for error reduction, once the error reaches to desired level of tolerance, training of data finally completed [34]. In the present study, the fuel blend, engine speed and loading conditions were selected as input factors of the input layer. The performance and emission characteristics (Torque, BP, BSFC, BTE CO, CO_2_, HC, and NOx) were devoted to output layer. Total experimentally recorded observations were 144, which served as a dataset. The proposed ANN network structure is presented in Fig. 7. The ANN model comprised of three input nodes: fuel blend, engine speed and load, with sole hidden layer comprised of ten nodes, and output layer comprised of eight nodes (one neuron against each response). The model was created using the MATLAB NN Toolbox, which separated the input into three sets at random such that 70 % includes training, 15 % includes validation, and 15 % includes testing.

**Fig. 8:**
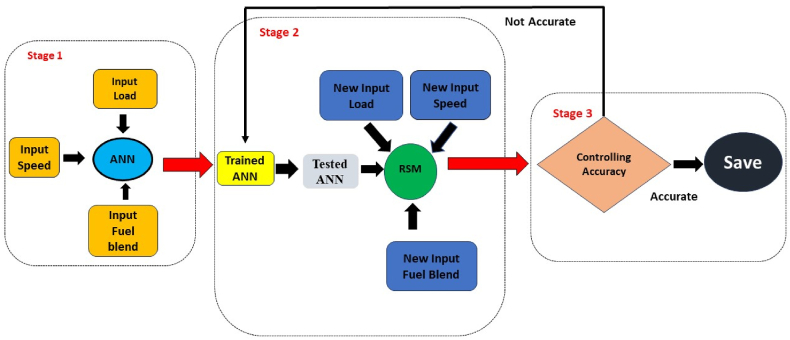
Combined working of ANN and RSM for accepted models.

**Fig. 9:**
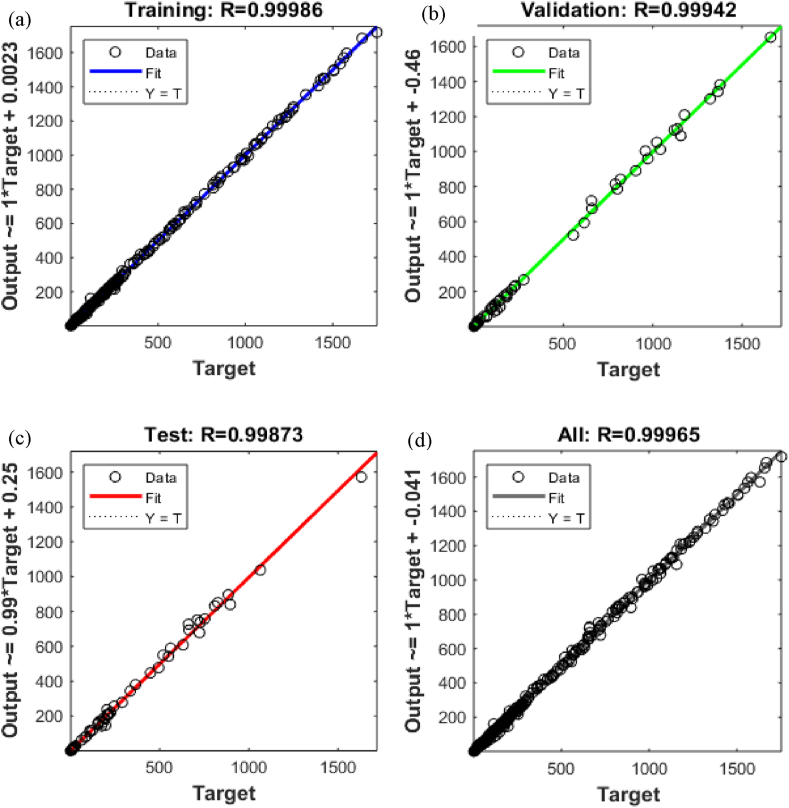
R values for (a) training, (b) validation, (c) testing data, and (d) all empirical results.

The feedforward backpropagation network in the ANN modelling was used due to its indispensable impact in the system modelling, data signal processing with accuracy and non-linear behavior [72]. The tansig transfer function is highly suitable for larger dataset with higher efficiency rate and quick learning rate. The LEARNGDM learning function was opted for reducing errors. The quantity of the neurons in hidden layer are crucial for an ANN model to function effectively; otherwise, there may be insufficient correlation among anticipated responses and input variables, leading to an inconsistent model [69,73]. The optimized model was selected based on least MRE between empirical and predicted responses, expressed in equation (1). The correlation coefficient (R) nearest to positive unity is usually attained for the best-predicted responses. It signifies the linearity among empirical and predicted response as positive and indicates highly accurate results [74]. As per set criterion, the training was stopped when validation error limit was surpassed. The current research utilizes 10 neurons, which is backed by extensive literature review. The criterion of the neurons however comes with uncertainty due to results which may be undesirable. The inappropriate selection of the learning conditions results in significant error difference between training and testing due to overfitting of trained ANN model. The comprehensive network architecture for both ANN and RSM operations is depicted in Fig. 8. The schematic chart in Fig. 9 illustrates the ANN operation. The various stages of ANN model show the specifications of input (1st stage), network training on parameters for curtailing discrepancies (2nd stage) and result validation based on input characteristics (3rd stage). R and MRE values served as key indicators to estimate results statistically. The benchmark of R greater than 0.99 and MRE less than 3 % was fixed as a key indicator for successful ANN model. In case, the set benchmark was not attained during thousand iterations for any response, an adaptation rate was then changed. The learning rate was fixed in between the step rise of 1.1 and step decline of 0.9. The responses of ANN model were tested through statistical measures of MRE, RMSE, and R^2^, as expressed in equations (1), (2), (3):

(1) $$MRE\left(\%\right)=\frac{1}{n}\sum_{i=1}^{n}\left|100\frac{T_{i}-P_{i}}{P_{i}}\right|$$

(2) $$RMSE=\sqrt{\frac{1}{n}\sum_{i=1}^{n}{\left(P_{i}-T_{i}\right)}^{2}}$$

(3) $$R^{2}=1-\left(\frac{\sum_{i=1}^{n}{\left(P_{i}-T_{i}\right)}^{2}}{\sum_{i=1}^{n}{\left(T_{i}\right)}^{2}}\right)$$

The prediction of the SI engine parameters for the distinct butanol blends using ANN model proved extremely successful. The first ANN model with overall regression graphs for all parameters is portrayed in Fig. 9(a–d). The ANN generated model produced highly precise results against each factor. The comparative assessment of empirical results with predicted responses produced an overall R value of 0.99965 and MRE less than 3 %. The R values for training, validation and testing were found to be 0.99986, 0.99942 and 0.99872 respectively. ANN predicted results met statistical criteria as demarcated in previous segments. After the success of the first ANN model, the ANN model then developed for the individual outputs for comprehensive prediction of values. The feed-forward back propagation algorithm and network structure generated satisfactorily adequate outcomes.

**Fig. 10:**
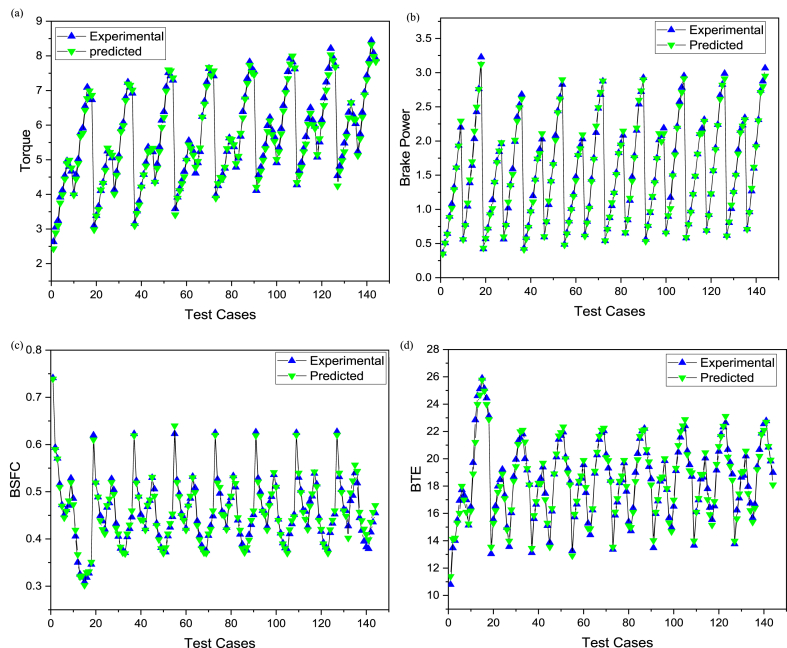
Analysis of ANN predicted and empirical results of (a) Torque, (b) Brake Power, (c) BSFC, and (d) BTE for each test case.

**Fig. 11:**
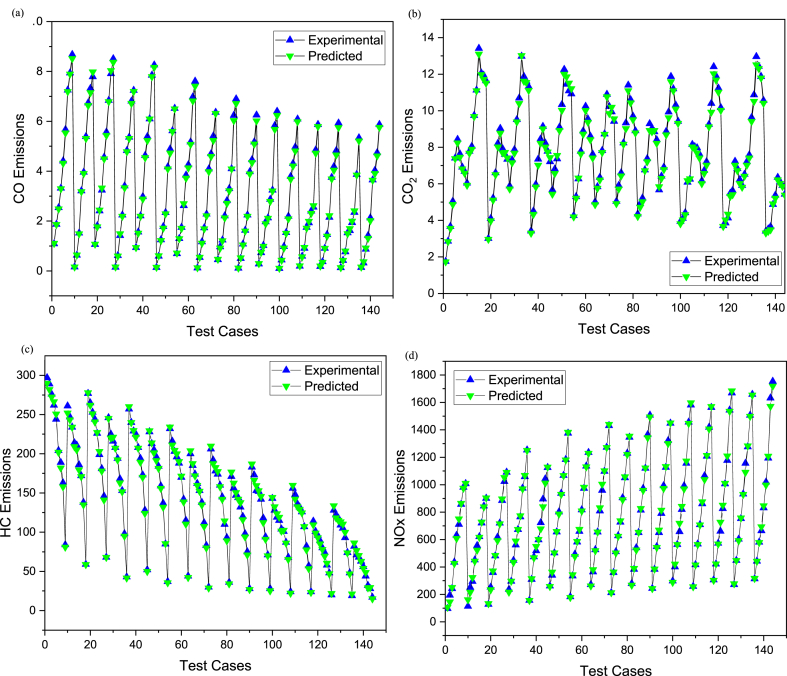
Analysis of ANN predicted and empirical results of (a) CO, (b) CO_2_, (c) HC, and (d) NOx for each test case.

**Fig. 12:**
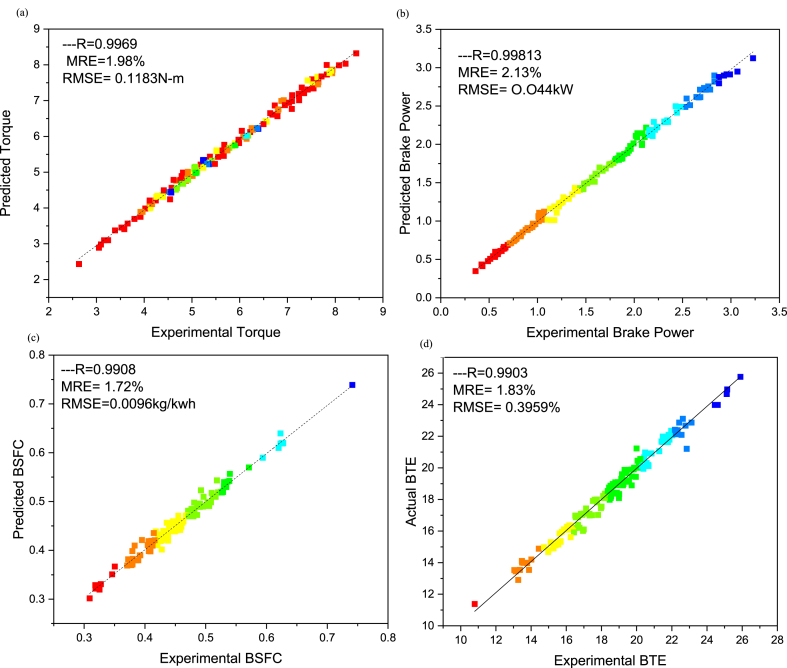
Comparison between ANN predicted and empirical results of (a) Torque, (b) Brake Power, (c) BSFC, and (d) BTE.

**Fig. 13:**
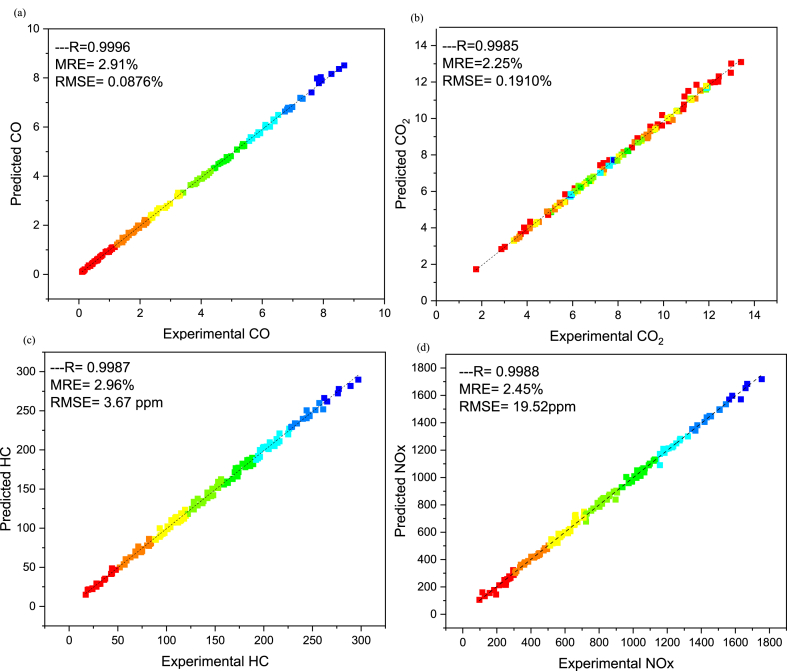
Comparison between ANN predicted and empirical results of (a) CO, (b) CO_2_, (c) HC, and (d) NOx.

The comparative assessment for empirical and predicted engine parameters are demonstrated in Fig. 12(a–d). The R value for predicted torque, BP, BSFC and BTE were 0.9969, 0.9981, 0.9908 and 0.9903 respectively. MREs were evaluated as 1.98 %, 2.13 %, 1.72 % and 1.83 % for torque, BP, BSFC and BTE respectively. The RMSEs were 0.1183 N-m, 0.044 kW, 0.0096 kg/kWh and 0.3959 % for torque, BP, BSFC and BTE respectively. The ANN predicted SI engines parameters for different butanol blended fuels produced extremely precise outcomes. ANN model prediction with MRE within range of 1.83–2.13 % and R values within 0.9903–0.9981 for all performance parameters. Likewise, the RMSE values were quite low for performance parameters of the engine. It signifies that the SI engines parameters can be precisely simulated through suitable ANN modelling. Fig. 10(a–d) denotes the comparison between the empirical and ANN predicted responses against 144 test cases for performance parameters. The comparative assessment for empirical and ANN predicted emission characteristics is demonstrated in Fig. 13(a–d). The R values for predicted CO, CO_2_, HC, and NOx were 0.9996, 0.9985, 0.9987 and 0.9988 respectively. MREs were calculated as 2.91 %, 2.25 %, 2.96 % and 2.45 % for CO, CO_2_, HC, and NOx respectively. The RMSEs were 0.0876 %, 0.1910 %, 3.67 ppm and 19.52 ppm for CO, CO_2_, HC, and NOx respectively. The ANN predicted SI engines parameters for different butanol blended fuels produced extremely precise outcomes. ANN model prediction with MRE within range of 2.25–2.96 % and R values within 0.9985–0.9996 for all emission parameters. Likewise, RMSE values were quite low for emission parameters of engine. It signifies that SI engines parameters can be precisely simulated through suitable ANN modelling. Fig. 11(a–d) denotes the comparison between empirical and ANN predicted responses against 144 test cases for CO, CO_2_, HC, and NOx respectively.

### 3.3 RSM based optimization

Process optimization aims to attain maximum output by maneuvering controlled variables. The numerical constraints play a key role in maximizing or minimizing the response variables in any optimization problem. The RSM is an eminent statistical technique employed for optimizing empirical data for solving simultaneous equations. The accurate response prediction through the RSM serves as the main reason for its use in the engineering sector. In the selected model, the performance and emission parameters of the test engine were designated as response variables. The goal was to enhance performance except BSFC and curtail exhaust emissions. The design factors accounted for optimization of engine performance were fuel blend (%), engine speed (rpm) and engine load (psi). Design Expert software was used for generating model and response surfaces. With the help of historical data characteristics, a multilayer design for a pre-defined empirical investigation was established. Table 5 includes three input factors, engine speed (nine levels), fuel blend (eight levels), and load (two levels). The levels of these numeric factors served as model classifying characteristics.

**Table 5:** Input factors with levels.

Factors	Units	Levels	L [1]	L [2]	L [3]	L [4]	L [5]	L [6]	L [7]	L [8]	L [9]
Fuel blend	%	8	0	3	6	9	12	15	18	21	
Load	psi	2	15	30							
Engine speed	rpm	9	1300	1600	1900	2200	2500	2800	3100	3400	3700

### 3.4 Empirical model selection

The fit summaries of performance and emission parameters are detailed in Table 6, Table 7, Table 8, Table 9. Usually, the choice of suitable model depends on (i) p-value, (ii) predicted R^2^, and (iii) reasonable compliance between predicted and adjusted R^2^ [75]. Depending upon the stated parameters, linear and 2FI models possess smaller R^2^ values. But the quadratic model exhibits the best fit, due to p-values less than 0.0001 and R^2^ considerably near to 1. Depending on the review of previous literature, complex engine combustion process can be appropriately explained through quadratic model [76]. That's why the quadratic model was opted for optimization setup.

**Table 6:** Torque and Brake power fit summary.

Source	Torque fit summary			Brake power fit summary		
	p-value	Adjusted R^2^	Predicted R^2^	p-value	Adjusted R^2^	Predicted R^2^
Linear	<0.0001	0.9210	0.9177	<0.0001	0.9622	0.9604
2FI	<0.0001	0.9421	0.9374	<0.0001	0.9869	0.9856
Quadratic	<0.0001	0.9747	0.9728	0.0051	0.9877	0.9862

**Table 7:** BSFC and BTE fit summary.

Source	BSFC fit summary			BTE fit summary		
	p-value	Adjusted R^2^	Predicted R^2^	p-value	Adjusted R^2^	Predicted R^2^
Linear	<0.0001	0.4861	0.4623	<0.0001	0.4785	0.4561
2FI	0.0173	0.5122	0.4674	0.0365	0.4991	0.4610
Quadratic	<0.0001	0.9058	0.8922	<0.0001	0.8948	0.8835

**Table 8:** CO and CO_2_ fit summary.

Source	CO fit summary			CO_2_ fit summary		
	p-value	Adjusted R^2^	Predicted R^2^	p-value	Adjusted R^2^	Predicted R^2^
Linear	<0.0001	0.9330	0.9303	<0.0001	0.3877	0.3617
2FI	<0.0001	0.9634	0.9610	<0.0001	0.8072	0.7943
Quadratic	<0.0001	0.9737	0.9716	<0.0001	0.9144	0.9077

**Table 9:** HC and NOx fit summary.

Source	HC fit summary			NOx fit summary		
	p-value	Adjusted R^2^	Predicted R^2^	p-value	Adjusted R^2^	Predicted R^2^
Linear	<0.0001	0.9090	0.9046	<0.0001	0.9597	0.9577
2FI	<0.0001	0.9373	0.9320	<0.0001	0.9788	0.9771
Quadratic	<0.0001	0.9767	0.9741	<0.0001	0.9827	0.9808

### 3.5 Analysis of Variance (ANOVA)

ANOVA is a statistical technique for evaluating interaction between factors along with the statistical significance of the model. It gives a comprehensive understanding of the regression models in order to comprehend the interactions between factors and responses. Table 10, Table 11, Table 12, Table 13, Table 14, Table 15, Table 16, Table 17 depict ANOVA for quadratic models in the case of performance and emission parameters. The F values of 775.47, 1469.86, 172.16, 151.64, 795.94, 257.40, 801.42, and 1226.31 for torque, BP, BSFC, BTE, CO, CO_2_, HC, NOx indicate the significance of models. The terms in the model were coded as A for fuel blend, B for load, and C for engine speed. The p values lower than 0.0860 also highlight that model terms are significant.

**Table 10:** ANOVA for torque.

Source	Sum of Squares	df	Mean Square	F-value	p-value
Model	243.90	7	34.84	775.47	<0.0001
A-Fuel Blend	31.50	1	31.50	701.07	<0.0001
B-Load	96.75	1	96.75	2153.38	<0.0001
C-Speed	105.11	1	105.11	2339.44	<0.0001
AB	0.6816	1	0.6816	15.17	0.0002
AC	0.3854	1	0.3854	8.58	0.0040
BC	4.06	1	4.06	90.28	<0.0001
C^2^	5.41	1	5.41	120.40	<0.0001

**Table 11:** ANOVA for Brake power.

Source	Sum of Squares	df	Mean Square	F-value	p-value
Model	76.12	6	12.69	1469.86	<0.0001
A-Fuel Blend	1.34	1	1.34	155.68	<0.0001
B-Load	7.92	1	7.92	917.47	<0.0001
C-Speed	64.99	1	64.99	7529.13	<0.0001
AB	0.1509	1	0.1509	17.48	<0.0001
BC	1.64	1	1.64	190.36	<0.0001
A^2^	0.0780	1	0.0780	9.04	0.0031

**Table 12:** ANOVA for BSFC.

Source	Sum of Squares	Df	Mean Square	F-value	p-value
Model	0.6442	7	0.0920	172.16	<0.0001
A-Fuel Blend	0.0100	1	0.0100	18.79	<0.0001
B-Load	0.2530	1	0.2530	473.30	<0.0001
C-Speed	0.0570	1	0.0570	106.67	<0.0001
AB	0.0173	1	0.0173	32.43	<0.0001
AC	0.0057	1	0.0057	10.65	0.0014
BC	0.0076	1	0.0076	14.28	0.0002
C^2^	0.2934	1	0.2934	548.97	<0.0001

**Table 13:** ANOVA for BTE.

Source	Sum of Squares	Df	Mean Square	F-value	p-value
Model	1039.34	8	129.92	151.64	<0.0001
A-Fuel Blend	1.26	1	1.26	1.47	0.2281
B-Load	511.16	1	511.16	596.64	<0.0001
C-Speed	36.47	1	36.47	42.57	<0.0001
AB	27.72	1	27.72	32.36	<0.0001
AC	10.03	1	10.03	11.70	0.0008
BC	3.21	1	3.21	3.74	0.0552
A^2^	3.17	1	3.17	3.70	0.0565
C^2^	446.33	1	446.33	520.97	<0.0001

**Table 14:** ANOVA for CO emission.

Source	Sum of Squares	df	Mean Square	F-value	p-value
Model	762.30	7	108.90	795.94	<0.0001
A-Fuel Blend	74.84	1	74.84	547.01	<0.0001
B-Load	7.02	1	7.02	51.28	<0.0001
C-Speed	646.85	1	646.85	4727.77	<0.0001
AB	10.27	1	10.27	75.03	<0.0001
AC	14.50	1	14.50	105.99	<0.0001
A^2^	0.9251	1	0.9251	6.76	0.0103
C^2^	7.91	1	7.91	57.78	<0.0001

**Table 15:** ANOVA for CO_2_ emission.

Source	Sum of Squares	df	Mean Square	F-value	p-value
Model	868.72	6	144.79	257.40	<0.0001
A-Fuel Blend	9.20	1	9.20	16.36	<0.0001
B-Load	1.17	1	1.17	2.07	0.1521
C-Speed	378.56	1	378.56	673.00	<0.0001
AB	385.30	1	385.30	684.98	<0.0001
AC	4.34	1	4.34	7.71	0.0063
C^2^	90.15	1	90.15	160.27	<0.0001

**Table 16:** ANOVA for HC emission.

Source	Sum of Squares	df	Mean Square	F-value	p-value
Model	6.771E+05	7	96724.79	801.42	<0.0001
A-Fuel Blend	2.804E+05	1	2.804E+05	2323.39	<0.0001
B-Load	34443.32	1	34443.32	285.38	<0.0001
C-Speed	3.168E+05	1	3.168E+05	2624.98	<0.0001
AC	17284.80	1	17284.80	143.21	<0.0001
BC	1906.10	1	1906.10	15.79	0.0001
A^2^	360.91	1	360.91	2.99	0.0860
C^2^	25851.87	1	25851.87	214.20	<0.0001

**Table 17:** ANOVA for NOx emission.

Source	Sum of Squares	df	Mean Square	F-value	p-value
Model	2.271E+07	7	3.244E+06	1226.31	<0.0001
A-Fuel Blend	2.132E+06	1	2.132E+06	806.22	<0.0001
B-Load	1.107E+05	1	1.107E+05	41.84	<0.0001
C-Speed	1.996E+07	1	1.996E+07	7546.96	<0.0001
AC	3.963E+05	1	3.963E+05	149.83	<0.0001
BC	10279.44	1	10279.44	3.89	0.0507
A^2^	44269.42	1	44269.42	16.74	<0.0001
C^2^	49446.37	1	49446.37	18.69	<0.0001

For torque, the F-value of 775.47 and p-value lower than of 0.0500 depict that model as significant. In this case A, B, C, AB, AC, BC, C^2^ are significant model terms. In the case of numerous insignificant model terms except those which support hierarchy, model reduction can improve the model. For brake power, the F-value of 1469.86 and p-value lower than of 0.0500 depict that model is significant. In this case A, B, C, AB, BC, A^2^ are significant model terms. For brake specific fuel consumption, the F-value of 172.16 and p-value lower than of 0.0500 depict that model is significant. In this case A, B, C, AB, AC, BC, C^2^ are significant model terms. For brake thermal efficiency, the F-value of 151.64 and p-value lower than of 0.0500 depict that model is significant. In this case B, C, AB, AC, C^2^ are significant model terms. In case of CO emission, the F-value of 795.94 and p-value lower than of 0.0500 depict that model is significant. In this case A, B, C, AB, AC, A^2^, C^2^ are significant model terms. In terms of the CO_2_ emission, the F-value of 257.40 and p-value lower than of 0.0500 depict that model is significant. In this case A, C, AB, AC, C^2^ are significant model terms. For HC emission, the F-value of 801.42 and p-value lower than of 0.0500 depict that model is significant. In this case A, B, C, AC, BC, C^2^ are significant model terms. In terms of NOx emission, the F-value of 1226.31 and p-value lower than of 0.0500 depict that model is significant. In this case A, B, C, AC, A^2^, C^2^ are significant model terms.

**Fig. 14:**
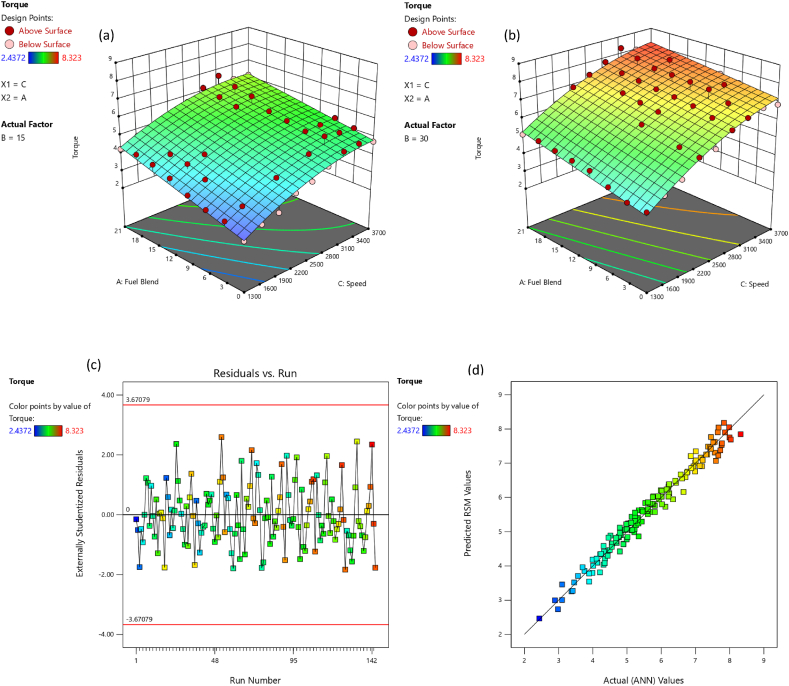
(a) Response surface for torque at lower load, (b) Response surface for torque at higher load, (c) Residual versus run graph for torque, and (d) Predicted versus actual graph for torque.

**Fig. 15:**
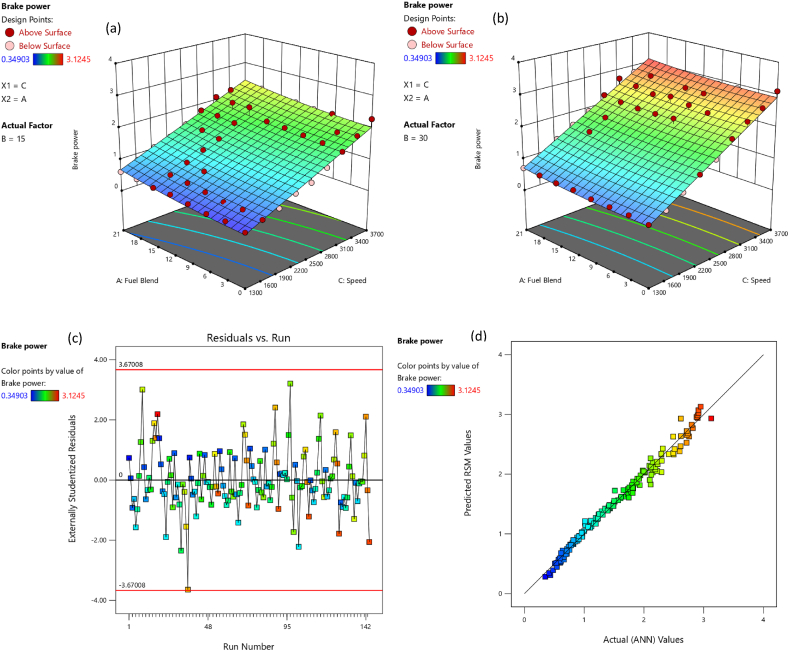
(a) Response surface for brake power at lower load, (b) Response surface for brake power at higher load, (c) Residual versus run graph for brake power, and (d) Predicted versus actual graph for brake power.

**Fig. 16:**
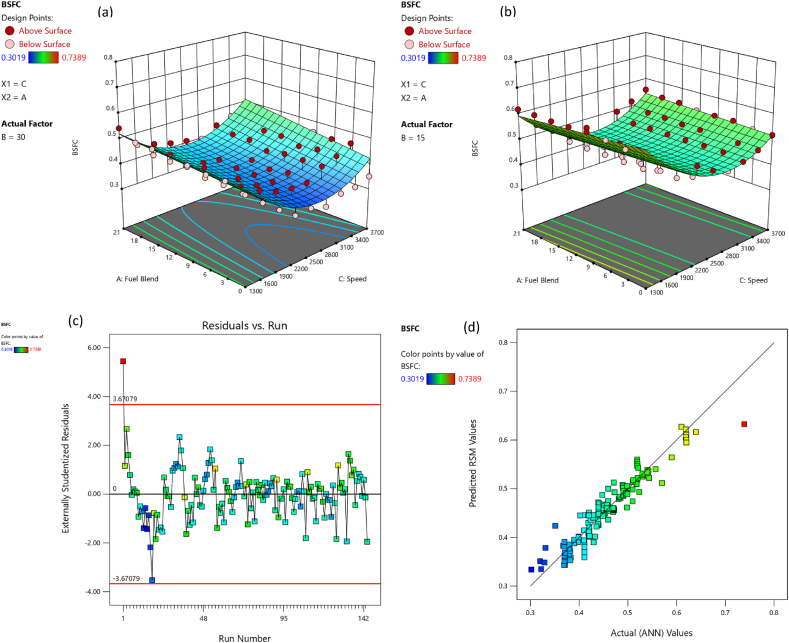
(a) Response surface for BSFC at lower load, (b) Response surface for BSFC at higher load, (c) Residual versus run graph for BSFC, and (d) Predicted versus actual graph for BSFC.

**Fig. 17:**
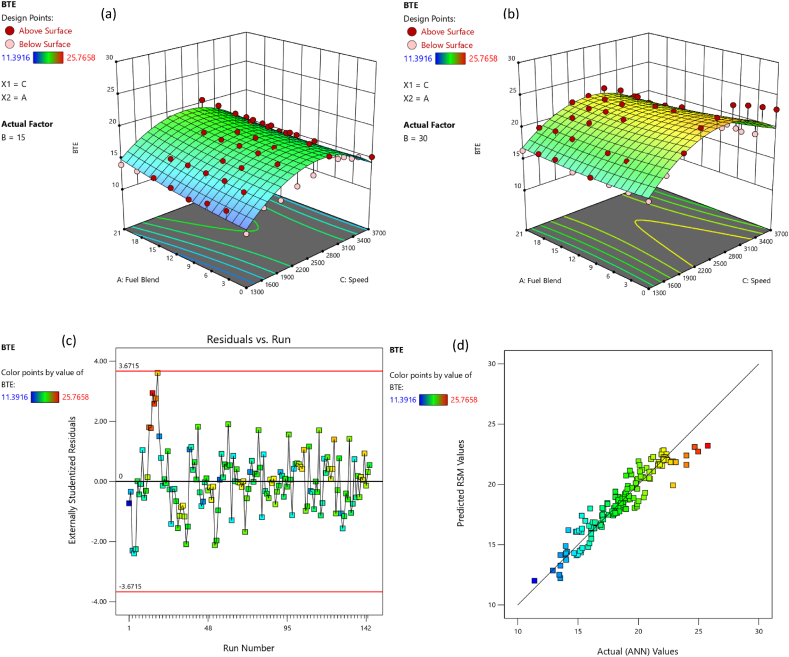
(a) Response surface for BTE at lower load, (b) Response surface for BTE at higher load, (c) Residual versus run graph for BTE, and (d) Predicted versus actual graph for BTE.

**Fig. 18:**
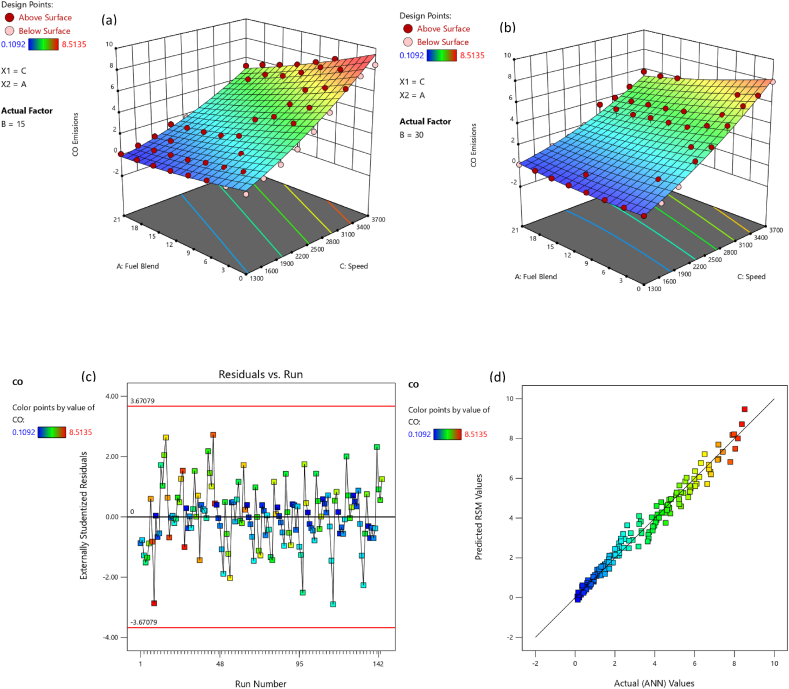
(a) Response surface for CO emission at lower load, (b) Response surface for CO emission at higher load, (c) Residual versus run graph for CO emission, and (d) Predicted versus actual graph for CO emission.

**Fig. 19:**
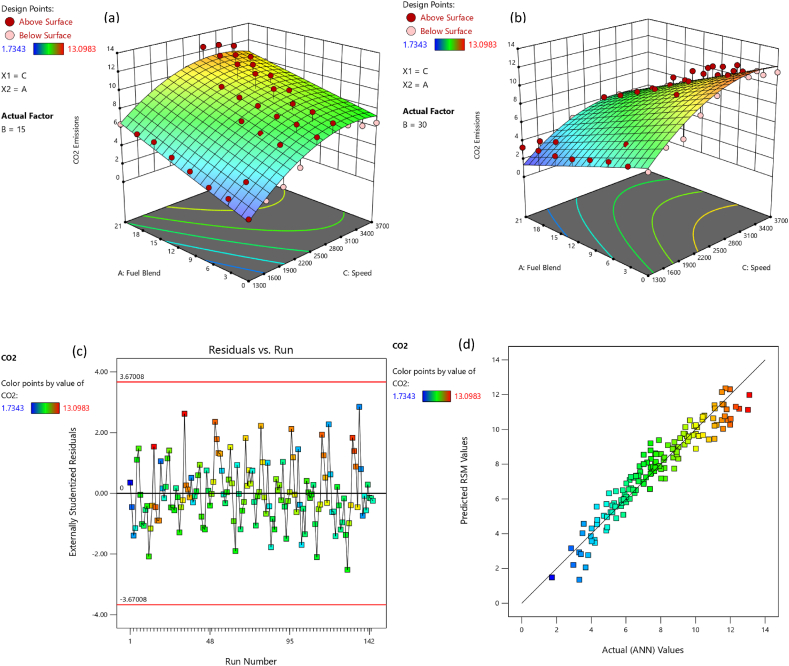
(a) Response surface for CO_2_ emission at lower load, (b) Response surface for CO_2_ emission at higher load, (c) Residual versus run graph for CO_2_ emission, and (d) Predicted versus actual graph for CO_2_ emission.

**Fig. 20:**
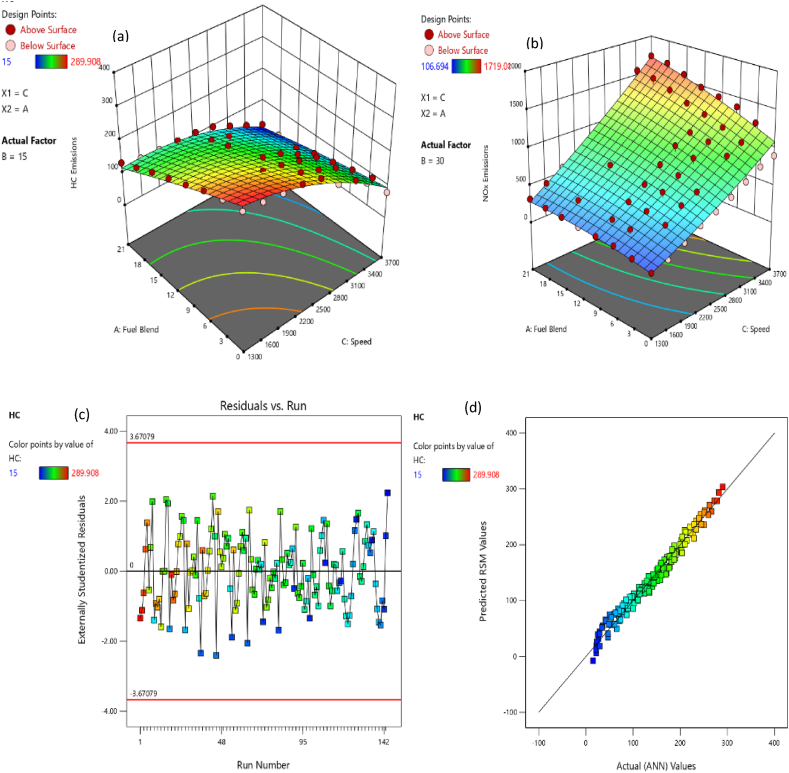
(a) Response surface for HC emission at lower load, (b) Response surface for HC emission at higher load, (c) Residual versus run graph for HC emission, and (d) Predicted versus actual graph for HC emission.

**Fig. 21:**
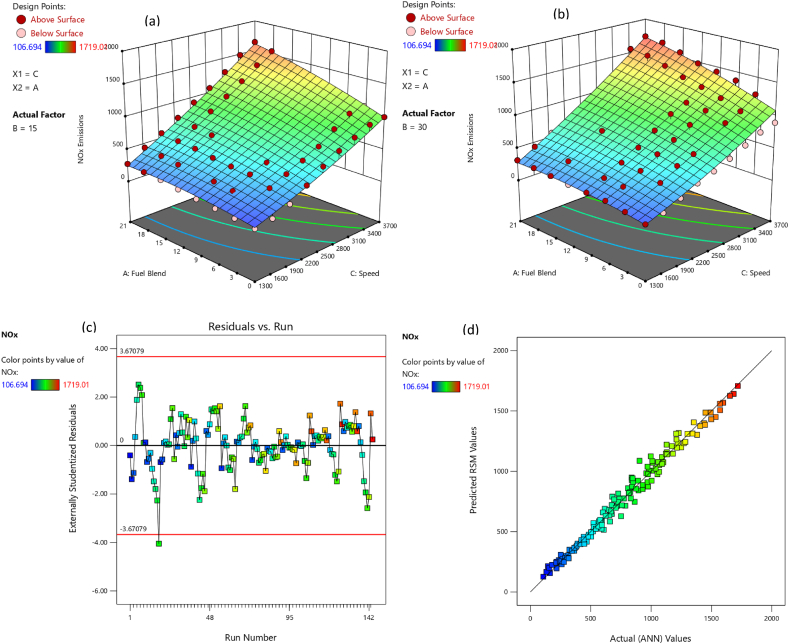
(a) Response surface for NOx emission at lower load, (b) Response surface for NOx emission at higher load, (c) Residual versus run graph for NOx emission, and (d) Predicted versus actual graph for NOx emission.

The adequacy of the considered models has been affirmed through residual versus run plots and diagnostic predicted versus actual as depicted in Fig. 14, Fig. 15, Fig. 16, Fig. 17, Fig. 18, Fig. 19, Fig. 20, Fig. 21 (c and d). Likewise, Fig. 14, Fig. 15, Fig. 16, Fig. 17, Fig. 18, Fig. 19, Fig. 20, Fig. 21 (a and b) depict the RSM predicted responses are close to ANN values, depicted by colored data points located near to linearly inclined line. The difference between RSM and actual (ANN) values was in between residual range of [−3.67, +3.67], as portraying Fig. 14, Fig. 15, Fig. 16, Fig. 17, Fig. 18, Fig. 19, Fig. 20, Fig. 21 (d). The uniform distribution at top and bottom of reference axis, for all parameters, indicates statistical significance of all (performance and emission parameters) RSM models. Response surfaces of torque, BP, BSFC, BTE, CO, CO_2_, HC and NO_X_ against engine speed, fuel blend, and load are portrayed in Fig. 14, Fig. 15, Fig. 16, Fig. 17, Fig. 18, Fig. 19, Fig. 20, Fig. 21 (a and b) respectively. It is evident that each design factor has a prominent impact on responses. The light and dark points on response surfaces indicates design points lower and higher than the forecasted values respectively. Regression equation (2nd order) associating the input factors and responses to evaluate performance and emissions are indicated by coded equations from 4 to 11. The coded alphabets A, B, and C represent the fuel blend, loading condition, and engine speed respectively. The equation including three coded input factors predicts the response against designated levels. As a default setting, factors with higher levels are represented as +1 and factors with low levels are represented as −1. The coded equation is beneficial for classifying relative effect of each factor through comparison between coefficients.

(4) $$\text{Torque}=5.82+0.7144A+0.7144A+0.8197B+1.32C\;–\;0.1051\text{AB}\;–\;0.1224\text{AC}+0.2600\text{BC}\;–0.5301C^{2}$$

(5) $$\text{Brake}\;\text{power}=1.53+0.1476A+0.2345B+1.04C\;–\;0.0494\text{AB}+0.1655\;\text{BC}+0.0622\;A^{2}$$

(6) $$\text{BSFC}=0.4062+0.0128A\;–0.0419B\;–\;0.0308C+0.0168\text{AB}+0.0149\text{AC}+0.0113\text{BC}+0.1235C^{2}$$

(7) $$\text{BTE}=20.30+0.1427A+1.88B+0.7796C\;–\;0.6702\text{AB}\;–\;0.6244\text{AC}+0.2311\text{BC}+0.3967A^{2\;}–\;4.82C^{2}$$

(8) $$\text{CO}=2.85\;–\;1.10A\;–\;0.2207B+3.28C+0.4078\text{AB}\;–\;0.7509\;\text{AC}+0.2143\;A^{2\;}+0.6408C^{2}$$

(9) $${\text{CO}}_{2}=8.59\;–\;0.3861A\;–\;0.0900B+2.51C\;–\;2.50\;\text{AB}\;–\;0.4107\text{AC}\;–\;2.16\;C^{2}$$

(10) $$\text{HC}=157.33\;–\;67.41A\;–\;15.47B\;–\;72.67C+25.93\text{AC}+5.64\;\text{BC}\;–\;4.23\;A^{2}\;–\;36.65\;C^{2\;}$$

(11) $$\text{NOx}=775.48+185.89A+27.27B+576.80C+124.15\text{AC}+13.09\;\text{BC}\;–\;46.87\;A^{2}+50.68\;C^{2}$$

### 3.6 Optimization and validation of results

The purpose of employing RSM in current investigation was to spot the optimum operating conditions for experimentation. The three design factors (fuel blend, engine speed and loading condition) were considered as input, and ANN predicted data (torque, BP, BSFC, BTE, CO, CO_2_, HC, and NOx) were used as response. An optimization function in design expert software requires optimal constrictions to be clear for design factors along with output responses. Table 18 depicts the designed constraints along with optimization setup. The objective was the optimization of the engine through maximizing its performance and minimizing exhaust emissions while setting the criteria of within range for study factors.

**Table 18:** Optimization setup.

Name	Goal	Lower Limit	Upper Limit	Lower Weight	Upper Weight	Importance
A: Fuel Blend	is in range	0	21	1	1	3
B: Load	is in range	15	30	1	1	3
C: Speed	is in range	1300	3700	1	1	3
Torque	maximize	2.4372	8.323	1	1	3
Brake power	maximize	0.34903	3.1245	1	1	1
BSFC	minimize	0.3019	0.7389	1	1	3
BTE	maximize	11.3916	25.7658	1	1	3
CO	minimize	0.1092	8.5135	1	1	3
CO_2_	minimize	1.7343	13.0983	1	1	3
HC	minimize	15	289.908	1	1	3
NOx	minimize	106.694	1719.01	1	1	3

**Fig. 22:**
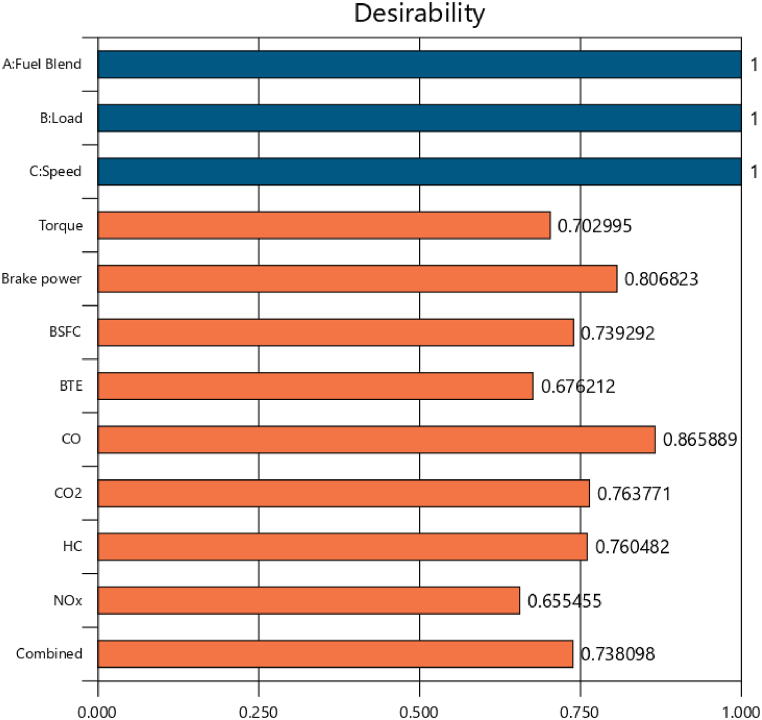
Desirability chart.

The most optimum operating condition came out to be 2000 rpm, B21, and 30psi loading condition. The performance characteristics against this optimum condition are 6.576 N-m torque, 1.437 kW brake power, 0.416 kg/kWh BSFC, 21.114 % of BTE, 1.237%v of CO emission, 4.421%v of CO_2_ emission, 80.840 ppm HC emission and 662.516 ppm NOx emission. The composite desirability (D) is a unitless quantity which varies from zero to one. The value closer to 1, the more effective optimization takes place. As the D was perceived to be 0.7381 (Fig. 22). A value adequately near to 1 proves the effectiveness of employed RSM models in predicting the optimum design factors for SI engine. The RSM based optimization can also be validated through empirical runs. The empirical findings of performance and emission parameters were noted consistent to optimized speed, fuel blend, and loads. The minimum and maximum individual desirability were observed for NOx emission (0.6553) and CO emission (0.8658). These numeric values indicate that varying CO emission will create max impact and varying NOx emission will create least impact on the setting as whole. The experimental observations indicate sufficient compliance with RSM optimized values with absolute percentage error (APE) below 4 % for all the performance and emission parameters (see Table 19). The lower APE between RSM predicted and experimental values depicts that the RSM is practically viable.

**Table 19:** APE between RSM predicted and experimental values.

Responses	Input factors (Blend (B21), Load (30 psi) and Speed (2000 rpm))		
	RSM Predicted	Experimental	APE
Torque (N-m)	7.248	7.485	3.27
Brake power (kW)	1.850	1.899	2.66
BSFC (kg/kWh)	0.389	0.401	3.21
BTE (%)	22.126	22.874	3.38
CO (%)	1.986	2.037	2.57
CO_2_ (%)	5.560	5.726	2.98
HC (ppm)	73.077	75.306	3.05
NOx (ppm)	870.834	897.829	3.10

### 3.7 ANN and RSM model comparison

The statistically dependent predicting models of performance parameters (Torque, brake power, BSFC and BTE) and emission parameters (CO, CO_2_, HC, and NOx) apparently exhibit alike efficiency and reliability. However, owing to general linkages of methods root task to similar domains, the comparative evaluation of these two models can be a perfect approach. A comprehensive comparison between the ANN and RSM models in terms of MRE and RMSE is exhibited in Table 20. It reveals that ANN model predicts engine characteristics more efficiently owing to lower values of RMSE and MRE.

**Table 20:** ANN and RSM comparison.

Parameters	ANN model		RSM model	
	MRE (%)	RMSE	MRE (%)	RMSE
Torque	1.98	0.1183 N-m	3.06	0.2060 N-m
Brake power	2.13	0.044 kW	2.99	0.091 kW
BSFC	1.72	0.0096 kg/kWh	3.61	0.022 kg/kWh
BTE	1.83	0.3959 %	3.65	0.8965 %
CO	2.91	0.0876 %v	3.01	0.0356 %v
CO_2_	2.25	0.1910 %v	2.99	0.2991 %v
HC	2.96	3.67 ppm	3.41	4.61 ppm
NOx	2.45	19.52 ppm	3.69	20.11 ppm

## 4 Compliance with Sustainable Development Goals (SDGs)

**Fig. 23:**
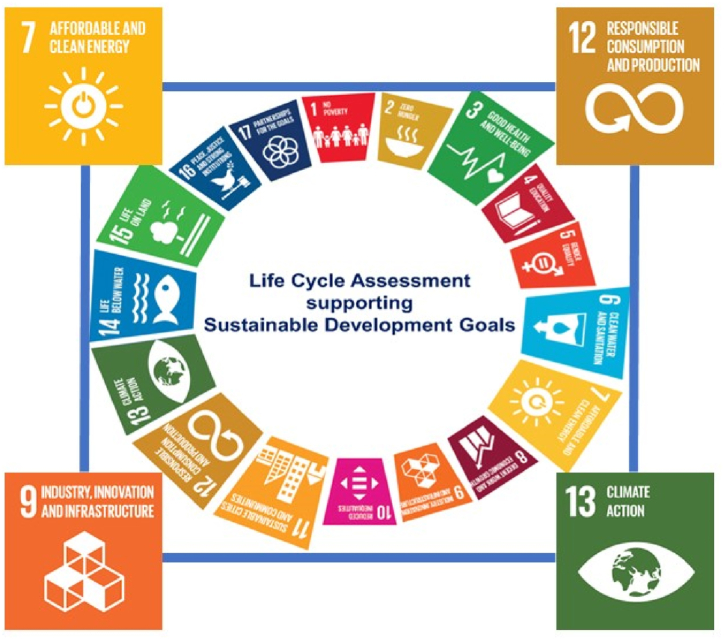
Link between current study and United Nations SDGs.

The exponential growth of industry and rapid utilization of resources without future planning has resulted in environmental havoc for all living beings. No field of science can remain insensitive towards the present climate challenges. Ignorance towards the environment has led to catastrophic change in climate. Different initiatives are taken by governments to preserve the climate. The American government has introduced a scrappage policy to encourage the customers to replace old and emission causing vehicles [77]. The ideology of the present study is in line with four points of the 2015 United Nation 17-point agenda of the Sustainable Development Goals (SDGs). It will also inspire the masses to responsibly produce and consume the resources to minimize emissions. The efforts of pollution reduction would help to preserve climate and sea life (13th SDG). The implementation of butanol as fuel in vehicles is directly under compliance with SDGs 7 (Affordable and Clean Energy) and 13 (Climate Action), which involve adoption of sustainable and clean energy sources to cope with climate change. SGD 7 signifies the need to switch from conventional petroleum sources to renewable sources by assisting the balance between climate change and industrialization promoting economic growth, and fulfilling energy demands [78,79]. The outcomes of the current study including both performance and exhaust emissions from the SI engine fueled with butanol blends are following SDGs 7 and 13. The unique combination of RSM and ANN for optimizing and predicting engine performance inspires effective resource utilization and sustainable production along with the establishment of better infrastructure facilities in order to harness natural sources. This approach is in alliance with SDGs 9 and 12 to promote inclusive and sustainable industrialization, and foster innovation along with sustainable consumption and production patterns. Fig. 23 represents the link between current study with SDGs under United Nations vision 2030.

## 5 Conclusions

The current study not only investigated the performance and emission characteristics of the SI engine fueled with butanol-gasoline blends, but also presented the comparative assessment of the ANN and RSM predicted results. The ANN and RSM techniques were subsequently employed for the performance and emissions prediction followed by optimization. The research outcomes are briefly explained as follows.

• The higher loading condition proved valuable in the experiment for assessment of engine performance parameters. The blends B0:B3:B21 at higher loading condition produced 50.22 %, 38.72 %, 40.99 %, 37.09 %, 38.46 %, 37.08 %, 28.79 %, and 26.44 %, more brake power than B0:B3:B21 at lower loading condition. The blends B0:B3:B21 at higher loading condition exhibit 7.71 %, 3.32 %, 3.33 %, 3.29 %, 3.30 %, 3.29 %, 3.17 %, and 3.16 % more BTE than B0:B3:B21 at lower loading condition.
• The blends B3 to B21 produced 7.42 %, 15.04 %, 25 %, 35.56 %, 44.52 %, 51.79 % and 59.36 % lower hydro-carbon emission than B0 respectively. While the blends B3, B6, B9, B12, B15, B18 and B21 produced 7.75 %, 13.32 %, 24.39 %, 35.15 %, 57.81 %, 56.44 % and 66.85 % higher NOx emission than B0 respectively.
• The correlation coefficients for training, validation and testing were found 0.99986, 0.99942 and 0.99872 respectively. ANN predicted responses were in accordance with statistical criterion. The R^2^ of torque, BSFC, BP, and BTE were 0.9969, 0.9908, 0.9981, and 0.9903 respectively. MREs were ascertained as 1.98 %, 1.72 %, 2.13 %, and 1.83 % for torque, BSFC, BP, and BTE respectively. The RMSEs were 0.1183 N-m, 0.044 kW, 0.3959 %, and 0.0096 kg/kWh against torque, BP, BTE and BSFC respectively.
• While the correlation coefficient (R^2^) for predicted results of CO, CO_2_, HC, and NOx were 0.9996, 0.9985, 0.9987 and 0.9988 respectively. The MREs were calculated as 2.91 %, 2.25 %, 2.96 % and 2.45 % and RMSEs were 0.0876 %, 0.1910 %, 3.67 ppm and 19.52 ppm for CO, CO_2_, HC, and NOx respectively.
• A multi-response optimization signified suitable optimization setting with D value of 0.7381. RSM predicted optimum condition of fuel blend (B21), engine speed (2000 rpm) and loading condition (30psi). The deviation between experimental results and RSM predicted results was below 4 %.
• The ANN predicted values for engine performance fueled with butanol blended fuel generated accurate results. ANN technique proved more accurate than RSM technique with MRE less than 3 % for all performance and emission parameters.
• The ability of ANNs to learn complex patterns and relationships from data can provide a competitive advantage in decision-making and automation. Developing and implementing robust neural network models may require substantial investment in terms of skilled personnel, computational resources, and data. However, the potential benefits in terms of improved efficiency, accuracy, and decision-making can outweigh the costs.
• RSM to systematically improve processes, reduce variability, and optimize product formulations. This can lead to cost savings, improved product quality, and increased efficiency in manufacturing. Implementing RSM may involve investments in experimentation, data collection, and statistical analysis. However, the potential return on investment comes from the improved understanding and control of processes, leading to more reliable and cost-effective production.

Thus, the blending of butanol into gasoline proved extremely valuable to enhance engine performance with lower emission levels. The statistical valuation means (R, MRE and RMSE) disclosed that the engine parameters (performance and emissions) can be precisely predicted by employing the ANN and RSM models. Categorically, butanol addition to gasoline is enviable for improved engine performance and to reduce dependency on fossil fuels. The blending can also be further optimized through Artificial Intelligence and statistical procedures. The current study has tried to evaluate the effect of the butanol blends on engine performance, but in the future their impacts on the lubricant oil inside engine with a stepwise increment in speed and load can be assessed through the ANN models with help of distinct training functions and algorithms. This will not only save time but also save a lot of money which will otherwise be spent on getting deteriorated lubricant oil for testing after specified hours of engine running. In summary, both ANN and RSM have commercial value in different domains. ANNs are powerful tools for complex pattern recognition and prediction tasks, while RSM is valuable for optimizing processes and improving outcomes in fields where experimentation and systematic analysis are critical. Integration of ANN and RSM with other emerging technologies like reinforcement learning, transfer learning, and quantum computing may shape the future landscape of AI, making systems more adaptive and capable.

## Data availability

Data will be made available on request.

## CRediT authorship contribution statement

**Muhammad Ali Ijaz Malik:** Writing – original draft, Software, Investigation, Formal analysis, Conceptualization. **Muhammad Usman:** Methodology, Visualization, Supervision, Conceptualization. **Muhammad Waqas Rafique:** Resources, Methodology. **Sohaib Raza:** Formal analysis, Data curation. **Muhammad Wajid Saleem:** Validation, Software. **Naseem Abbas:** Writing – review & editing. **Uzair Sajjad:** Formal analysis. **Khalid Hamid:** Writing – review & editing, Visualization, Project administration. **Mohammad Rezaul Karim:** Methodology, Resources. **Md Abul Kalam:** Writing – review & editing, Supervision, Conceptualization.

## Declaration of competing interest

The authors declare that they have no known competing financial interests or personal relationships that could have appeared to influence the work reported in this paper.

## References

[1] Ahmad T., Zhang D. (2020). A critical review of comparative global historical energy consumption and future demand: the story told so far. Energy Rep..

[2] Asghar M.M., Wang Z., Wang B., Zaidi S.A.H. (2020). Nonrenewable energy—environmental and health effects on human capital: empirical evidence from Pakistan. Environ. Sci. Pollut. Control Ser..

[3] Yun-shan G., Shah A., Chao H., Baluch A. (2009). Effect of biodiesel on the performance and combustion parameters of a turbocharged compression ignition engine. Pakistan J. Eng. Appl. Sci..

[4] Ijaz Malik M.A. (2023). Response surface methodology application on lubricant oil degradation, performance, and emissions in SI engine: a novel optimization of alcoholic fuel blends. Sci. Prog..

[5] Liu Z., Zuo Q., Wu G., Li Y. (2018). An artificial neural network developed for predicting of performance and emissions of a spark ignition engine fueled with butanol–gasoline blends. Adv. Mech. Eng..

[6] Zhu Y., Chen Z., Liu J. (2014). Emission, efficiency, and influence in a diesel n-butanol dual-injection engine. Energy Convers. Manag..

[7] Harrison J., Amezaga J., Boyes S. (2010). 7th International Biofuels Conference.

[8] Li Y. (2017). Experimental investigation of a spark ignition engine fueled with acetone-butanol-ethanol and gasoline blends. Energy.

[9] Zhang Z. (2018). Effects of fatty acid methyl esters proportion on combustion and emission characteristics of a biodiesel fueled marine diesel engine. Energy Convers. Manag..

[10] El-Seesy A.I., Waly M.S., Nasser A., El-Zoheiry R.M. (2022). Improvement of the combustion, emission, and stability features of diesel-methanol blends using n-decanol as cosolvent. Sci. Rep..

[11] Zhang Z. (2022). Performance, combustion and emission characteristics investigations on a diesel engine fueled with diesel/ethanol/n-butanol blends. Energy.

[12] Li Y., Gong J., Yuan W., Fu J., Zhang B., Li Y. (2017). Experimental investigation on combustion, performance, and emissions characteristics of butanol as an oxygenate in a spark ignition engine. Adv. Mech. Eng..

[13] Zhong Y., Han W., Jin C., Tian X., Liu H. (2022). Study on effects of the hydroxyl group position and carbon chain length on combustion and emission characteristics of Reactivity Controlled Compression Ignition (RCCI) engine fueled with low-carbon straight chain alcohols. Energy.

[14] Thakur A.K., Kaviti A.K., Singh R., Gehlot A. (2020). An overview of butanol as compression ignition engine fuel. Int. J. Energy a Clean Environ. (IJECE).

[15] Masum B., Kalam M., Masjuki H., Palash S., Fattah I.R. (2014). Performance and emission analysis of a multi cylinder gasoline engine operating at different alcohol–gasoline blends. RSC Adv..

[16] Nguyen D.D., Moghaddam H., Pirouzfar V., Fayyazbakhsh A., Su C.-H. (2021). Improving the gasoline properties by blending butanol-Al2O3 to optimize the engine performance and reduce air pollution. Energy.

[17] Yousif I.E., Saleh A.M. (2023). Butanol-gasoline blends impact on performance and exhaust emissions of a four stroke spark ignition engine. Case Stud. Therm. Eng..

[18] Deng X., Li J., Liang Y., Yang W. (2023). Dual-fuel engines fueled with n-butanol/n-octanol and n-butanol/DNBE: a comparative study of combustion and emissions characteristics. Energy.

[19] Hananto A.L. (2023). Elman and cascade neural networks with conjugate gradient polak-ribière restarts to predict diesel engine performance and emissions fueled by butanol as sustainable biofuel. Results in Engineering.

[20] Li Y. (2016). Combustion, performance and emissions characteristics of a spark-ignition engine fueled with isopropanol-n-butanol-ethanol and gasoline blends. Fuel.

[21] Karthikeya Sharma T., Amba Prasad Rao G., Madhu Murthy K. (2015). Effect of swirl on performance and emissions of CI engine in HCCI mode. J. Braz. Soc. Mech. Sci. Eng..

[22] Singh S.B., Dhar A., Agarwal A.K. (2015). Technical feasibility study of butanol–gasoline blends for powering medium-duty transportation spark ignition engine. Renew. Energy.

[23] Elfasakhany A. (2016). Experimental study of dual n-butanol and iso-butanol additives on spark-ignition engine performance and emissions. Fuel.

[24] Gu X., Huang Z., Cai J., Gong J., Wu X., Lee C.-f. (2012). Emission characteristics of a spark-ignition engine fuelled with gasoline-n-butanol blends in combination with EGR. Fuel.

[25] Shirazi S.A., Abdollahipoor B., Windom B., Reardon K.F., Foust T.D. (2020). Effects of blending C3-C4 alcohols on motor gasoline properties and performance of spark ignition engines: a review. Fuel Process. Technol..

[26] Balki M.K., Sayin C., Canakci M. (2014). The effect of different alcohol fuels on the performance, emission and combustion characteristics of a gasoline engine. Fuel.

[27] Mittal N., Athony R.L., Bansal R., Kumar C.R. (2013). Study of performance and emission characteristics of a partially coated LHR SI engine blended with n-butanol and gasoline. Alex. Eng. J..

[28] Elfasakhany A. (2014). Experimental study on emissions and performance of an internal combustion engine fueled with gasoline and gasoline/n-butanol blends. Energy Convers. Manag..

[29] Yusuf A.A., Inambao F.L., Farooq A.A. (2020). Impact of n-butanol-gasoline-hydrogen blends on combustion reactivity, performance and tailpipe emissions using TGDI engine parameters variation. Sustain. Energy Technol. Assessments.

[30] Huynh T.T., Le M.D., Duong D.N. (2019). Effects of butanol–gasoline blends on SI engine performance, fuel consumption, and emission characteristics at partial engine speeds. International Journal of Energy and Environmental Engineering.

[31] Asrar Hussain S.K., Usman M., Umer J., Farooq M., Noor F., Anjum R. (2022). A novel analysis of n-butanol–gasoline blends impact on spark ignition engine characteristics and lubricant oil degradation. Energy Sources, Part A Recovery, Util. Environ. Eff..

[32] Ahmed E., Usman M., Anwar S., Ahmad H.M., Nasir M.W., Malik M.A.I. (2021). Application of ANN to predict performance and emissions of SI engine using gasoline-methanol blends. Sci. Prog..

[33] Yap W.K., Ho T., Karri V. (2012). Exhaust emissions control and engine parameters optimization using artificial neural network virtual sensors for a hydrogen-powered vehicle. Int. J. Hydrogen Energy.

[34] Kiani M.K.D., Ghobadian B., Tavakoli T., Nikbakht A., Najafi G. (2010). Application of artificial neural networks for the prediction of performance and exhaust emissions in SI engine using ethanol-gasoline blends. Energy.

[35] Kapusuz M., Ozcan H., Yamin J.A. (2015). Research of performance on a spark ignition engine fueled by alcohol–gasoline blends using artificial neural networks. Appl. Therm. Eng..

[36] Ardebili S.M.S., Solmaz H., Mostafaei M. (2019). Optimization of fusel oil–Gasoline blend ratio to enhance the performance and reduce emissions. Appl. Therm. Eng..

[37] Usman M. (2021). Artificial neural network led optimization of oxyhydrogen hybridized diesel operated engine. Sustainability.

[38] Ramesh K., Alwarsamy T., Jayabal S. (2015). Prediction of cutting process parameters in boring operations using artificial neural networks. J. Vib. Control.

[39] Ghobadian B., Rahimi H., Nikbakht A., Najafi G., Yusaf T. (2009). Diesel engine performance and exhaust emission analysis using waste cooking biodiesel fuel with an artificial neural network. Renew. Energy.

[40] Baranitharan P., Ramesh K., Sakthivel R. (2019). Measurement of performance and emission distinctiveness of Aegle marmelos seed cake pyrolysis oil/diesel/TBHQ opus powered in a DI diesel engine using ANN and RSM. Measurement.

[41] Rajamohan S. (2022). Optimization of operating parameters for diesel engine fuelled with bio-oil derived from cottonseed pyrolysis. Sustain. Energy Technol. Assessments.

[42] Krishnamoorthi M., Malayalamurthi R., Sakthivel R. (2019). Optimization of compression ignition engine fueled with diesel-chaulmoogra oil-diethyl ether blend with engine parameters and exhaust gas recirculation. Renew. Energy.

[43] Liao S., Jiang D., Huang Z., Zeng K. (2006). Characterization of laminar premixed methanol–air flames. Fuel.

[44] Elfasakhany A., Mahrous A.-F. (2016). Performance and emissions assessment of n-butanol–methanol–gasoline blends as a fuel in spark-ignition engines. Alex. Eng. J..

[45] Schifter I., Diaz L., Gómez J., Gonzalez U. (2013). Combustion characterization in a single cylinder engine with mid-level hydrated ethanol–gasoline blended fuels. Fuel.

[46] Hsieh W.-D., Chen R.-H., Wu T.-L., Lin T.-H. (2002). Engine performance and pollutant emission of an SI engine using ethanol–gasoline blended fuels. Atmos. Environ..

[47] Ingamells J.C., Lindquist R. (1975). Methanol as a motor fuel or a gasoline blending component. SAE Trans..

[48] Broustail G., Seers P., Halter F., Moréac G., Mounaïm-Rousselle C. (2011). Experimental determination of laminar burning velocity for butanol and ethanol iso-octane blends. Fuel.

[49] Masum B., Masjuki H.H., Kalam M., Palash S., Habibullah M. (2015). Effect of alcohol–gasoline blends optimization on fuel properties, performance and emissions of a SI engine. J. Clean. Prod..

[50] Ijaz Malik M.A., Usman M., Hayat N., Zubair S.W.H., Bashir R., Ahmed E. (2021). Experimental evaluation of methanol-gasoline fuel blend on performance, emissions and lubricant oil deterioration in SI engine. Adv. Mech. Eng..

[51] Usman M. (2023). Comparative assessment of ethanol and methanol–ethanol blends with gasoline in SI engine for sustainable development. Sustainability.

[52] Bilgin A., Sezer I. (2008). Effects of methanol addition to gasoline on the performance and fuel cost of a spark ignition engine. Energy Fuel..

[53] Shayan S.B., Seyedpour S., Ommi F., Moosavy S., Alizadeh M. (2011). Impact of methanol–gasoline fuel blends on the performance and exhaust emissions of a SI engine. International Journal of Automotive Engineering.

[54] Elfasakhany A. (2017). Investigations on performance and pollutant emissions of spark-ignition engines fueled with n-butanol–, isobutanol–, ethanol–, methanol–, and acetone–gasoline blends: a comparative study. Renew. Sustain. Energy Rev..

[55] Varol Y., Öner C., Öztop H., Altun Ş. (2014). Comparison of methanol, ethanol, or n-butanol blending with unleaded gasoline on exhaust emissions of an SI engine. Energy Sources, Part A Recovery, Util. Environ. Eff..

[56] Tian Z., Zhen X., Wang Y., Liu D., Li X. (2020). Combustion and emission characteristics of n-butanol-gasoline blends in SI direct injection gasoline engine. Renew. Energy.

[57] Bayindir H., Yücesu H.S., Aydin H. (2010). The effects of λ and∊ on engine performance and exhaust emissions using ethanol–unleaded gasoline blends in an SI engine. Energy Sources, Part A Recovery, Util. Environ. Eff..

[58] Tian Z., Zhen X., Wang Y., Liu D., Li X. (2020). Comparative study on combustion and emission characteristics of methanol, ethanol and butanol fuel in TISI engine. Fuel.

[59] Yang J., Wang Y., Feng R. (2011).

[60] Szwaja S., Naber J.D. (2010). Combustion of n-butanol in a spark-ignition IC engine. Fuel.

[61] Masum B., Kalam M., Masjuki H., Rahman S.A., Daggig E. (2014). Impact of denatured anhydrous ethanol–gasoline fuel blends on a spark-ignition engine. RSC Adv..

[62] Abdalla A.N., Awad O.I., Tao H., Ibrahim T.K., Mamat R., Hammid A.T. (2019). Performance and emissions of gasoline blended with fusel oil that a potential using as an octane enhancer. Energy Sources, Part A Recovery, Util. Environ. Eff..

[63] Canakci M., Ozsezen A.N., Alptekin E., Eyidogan M. (2013). Impact of alcohol–gasoline fuel blends on the exhaust emission of an SI engine. Renew. Energy.

[64] Usman M. (2023). Acetone–gasoline blend as an alternative fuel in SI engines: a novel comparison of performance, emission, and lube oil degradation. ACS Omega.

[65] Veza I., Said M.F.M., Latiff Z.A. (2019). Progress of acetone-butanol-ethanol (ABE) as biofuel in gasoline and diesel engine: a review. Fuel Process. Technol..

[66] Dernotte J., Mounaïm-Rousselle C., Halter F., Seers P. (2010). Evaluation of butanol–gasoline blends in a port fuel-injection, spark-ignition engine. Oil & Gas Science and Technology–Revue de l’Institut Français du Pétrole.

[67] Wu G., Wu D., Li Y., Meng L. (2020). Effect of acetone-n-butanol-ethanol (ABE) as an oxygenate on combustion, performance, and emission characteristics of a spark ignition engine. J. Chem..

[68] Zaharin M.S.M., Abdullah N.R., Masjuki H.H., Ali O.M., Najafi G., Yusaf T. (2018). Evaluation on physicochemical properties of iso-butanol additives in ethanol-gasoline blend on performance and emission characteristics of a spark-ignition engine. Appl. Therm. Eng..

[69] Kesgin U. (2004). Genetic algorithm and artificial neural network for engine optimisation of efficiency and NOx emission. Fuel.

[70] Çay Y., Korkmaz I., Çiçek A., Kara F. (2013). Prediction of engine performance and exhaust emissions for gasoline and methanol using artificial neural network. Energy.

[71] Atik K., Kahraman N., Ceper B. (2013). Prediction of performance and emission parameters of an SI engine by using artificial neural networks. ISI BILIMI VE TEKNIGI DERGISI-JOURNAL OF THERMAL SCIENCE AND TECHNOLOGY.

[72] Kshirsagar C.M., Anand R. (2017). Artificial neural network applied forecast on a parametric study of Calophyllum inophyllum methyl ester-diesel engine out responses. Appl. Energy.

[73] Sayin C., Ertunc H.M., Hosoz M., Kilicaslan I., Canakci M. (2007). Performance and exhaust emissions of a gasoline engine using artificial neural network. Appl. Therm. Eng..

[74] Nasr G., Badr E., Joun C. (2003). Backpropagation neural networks for modeling gasoline consumption. Energy Convers. Manag..

[75] Abdalla A.N. (2019). Prediction of emissions and performance of a gasoline engine running with fusel oil–gasoline blends using response surface methodology. Fuel.

[76] Dey S., Reang N.M., Das P.K., Deb M. (2021). Comparative study using RSM and ANN modelling for performance-emission prediction of CI engine fuelled with bio-diesohol blends: a fuzzy optimization approach. Fuel.

[77] Li S., Liu Y., Wei C. (2022). The cost of greening stimulus: a dynamic analysis of vehicle scrappage programs. Int. Econ. Rev..

[78] Organization W.H. (2016).

[79] Zhao W., Zhang Y., Huang G., He Z., Qian Y., Lu X. (2021/12/01/2021). Experimental investigation on combustion and emission characteristics of butanol/biodiesel under blend fuel mode, dual fuel RCCI and ICCI modes. Fuel.

